# The effects of shared information on semantic calculations in the gene ontology

**DOI:** 10.1016/j.csbj.2017.01.009

**Published:** 2017-01-30

**Authors:** Paul W. Bible, Hong-Wei Sun, Maria I. Morasso, Rasiah Loganantharaj, Lai Wei

**Affiliations:** a State Key Laboratory of Ophthalmology, Zhongshan Ophthalmic Center, Sun Yat-sen University, Guangzhou 510060, China; b Biodata Mining and Discovery Section, Office of Science and Technology, Intramural Research Program, National Institute of Arthritis and Musculoskeletal and Skin Diseases, Bethesda, Maryland; c Laboratory of Skin Biology, Intramural Research Program, National Institute of Arthritis and Musculoskeletal and Skin Diseases, Bethesda, Maryland; d Laboratory of Bioinformatics, Center for Advanced Computer Studies, University of Louisiana at Lafayette, Lafayette, Louisiana

**Keywords:** Semantic similarity, Gene ontology, Function prediction, Machine learning, Protein–protein interaction, Gene expression

## Abstract

The structured vocabulary that describes gene function, the gene ontology (GO), serves as a powerful tool in biological research. One application of GO in computational biology calculates semantic similarity between two concepts to make inferences about the functional similarity of genes. A class of term similarity algorithms explicitly calculates the shared information (SI) between concepts then substitutes this calculation into traditional term similarity measures such as Resnik, Lin, and Jiang-Conrath. Alternative SI approaches, when combined with ontology choice and term similarity type, lead to many gene-to-gene similarity measures. No thorough investigation has been made into the behavior, complexity, and performance of semantic methods derived from distinct SI approaches. We apply bootstrapping to compare the generalized performance of 57 gene-to-gene semantic measures across six benchmarks. Considering the number of measures, we additionally evaluate whether these methods can be leveraged through ensemble machine learning to improve prediction performance. Results showed that the choice of ontology type most strongly influenced performance across all evaluations. Combining measures into an ensemble classifier reduces cross-validation error beyond any individual measure for protein interaction prediction. This improvement resulted from information gained through the combination of ontology types as ensemble methods within each GO type offered no improvement. These results demonstrate that multiple SI measures can be leveraged for machine learning tasks such as automated gene function prediction by incorporating methods from across the ontologies. To facilitate future research in this area, we developed the GO Graph Tool Kit (GGTK), an open source C++ library with Python interface (github.com/paulbible/ggtk).

## 1 Introduction

Researchers developed the gene ontology (GO) to provide a structured vocabulary that consistently describes the characteristics of genes and proteins across different organisms [1], [2]. Specific GO terms in this vocabulary annotate proteins by specifying the biological processes in which they participate, their enzymatic and molecular functions, and their location within the cell. As a structured vocabulary, GO explicitly defines the relationships between terms using a directed acyclic graph (DAG). These relationships serve to clarify terminology, for example by identifying when one term may be a more specialized from of another. Three separate ontologies exist that provide a DAG of terms and relationships used to describe biological processes (BP), molecular functions (MF), and cellular components (CC). These term structures are not fixed. The Gene Ontology Consortium makes frequent updates to GO modifying the relationship structure and adding or removing terms to better reflect the current understanding of biological functions.

The annotation of gene products with GO terms provides a valuable resource allowing the comparison of functions both within and between separate organisms. For each annotation of a term to a protein, GO provides evidence codes that allow researchers to consider the methods that produced each annotation. The use of GO plays an important role in the analysis of high-throughput experiments thanks in part to computational methods that utilize the rich domain knowledge encoded in GO annotations. The rigid, well-defined structure of GO proves to be an advantage that facilitates its integration into statistical and computational analyses. Methods of semantic similarity take advantage of this structure to quantify the similarity between the meaning of one term and another. Through semantic measures, the concept level knowledge stored in functional annotations provides the ability to quantify functional similarity between genes. Researchers have employed semantic methods for predicting protein–protein interactions [3], [4], [5], [6], prioritizing host-pathogen interactions [7], and automated function prediction [8], [9]. Increasingly, researchers apply computational methods to infer new GO annotations. These annotations receive the evidence code *inferred from electronic annotation* (IEA), and recent studies show that these predicted annotations are increasingly reliable [7], [10].

Since the seminal works by Lord et al. [11], [12], semantic similarity applications have become established tools in computational biology and bioinformatics. Many diverse methods exist for calculating semantic similarity between terms. The reviews of Refs. [13], [14], and [15] provide a thorough overview of semantic measures used in the Gene Ontology and other biomedical ontologies. The review by Pesquita et al. [13] categorizes semantic similarity at the term level into edge-based and node-based methods. Edge-based methods usually quantify semantic similarity using a function of the paths between two terms in the graph. Node-based methods use properties derived from the terms and often include operations on the shared ancestors or descendants of two terms. Lord et al. adapted three well studied information theoretic semantic measures, Resnik [16], Lin [17], and Jiang-Conrath [18], using a corpus-based calculation of term probability and information content (IC). These methods calculate the similarity between two concepts by operating on their shared and unique information. Lord et al. calculated shared information using the IC of the most informative common ancestor (MICA) between two terms. Information content based semantic methods have been extensively studied and research suggests that IC offers a superior conception of a term's specificity over methods based on graph depth [14], [19].

Couto et al. [20] developed an alternative approach to shared information. While the MICA shared information considers only a single ancestor, the alternative approach, called the graph-based similarity measure (GraSM), considers multiple inheritance of ontology terms using a path counting method. GraSM calculates the mean IC of disjunctive common ancestors between two concepts. Couto et al. recognized the modularity of using alternative shared information methods and substituted GraSM shared information into Resnik, Lin, and Jiang-Conrath deriving three new term similarity algorithms. This method was shown to improve the performance of semantic similarity on accepted evaluation metrics such as correlation with sequence and domain similarity. The computational cost of path counting poses a significant challenge to the real-time calculation of GraSM. Zhang and Lai [21] proposed a faster GraSM alternative called exclusively inherited shared information that calculates a subset of the disjunctive common ancestors. The modular separation of shared information from term similarity leads to some interesting properties. Any new conception of shared information immediately implies the construction of three new term similarity measures. The success of GraSM and alternative conceptions of shared information clarifies the need for a thorough exploration of shared information in the biomedical ontologies.

Extending *term* similarity to *gene* similarity requires methods operating on term sets. For two genes, represented as term sets, the all-pairs term similarity is calculated then summarized by aggregation methods such as the *min* [22], *max* [23], *average* [11], or *best-match average* (BMA) [24]. See Ref. [13] for a detailed description of aggregation methods. Furthermore, genes are described by annotations from each of the independent ontologies (BP, MF, CC). The choices for shared information calculation, term similarity algorithm, aggregation method, and ontology type lead to a combinatorial increase in the number of gene similarity measures. Considering the number of measure that can be constructed, we address the following questions in this work. Are the methods derived from alternative shared information calculations truly distinct or do they offer no statical difference in practical applications? If these methods are not distinct, it would imply that computationally intensive algorithms, such as GraSM, can be replaced by more efficient alternatives. Some gene similarity methods avoid explicit calculation of shared information, term similarity, and aggregation. Methods based on the simple yet powerful Jaccard set similarity, such as SimUI [25], SimGIC [19], SimDIC [26], and SimUIC [26], have been shown to perform well on a variety of tasks and are computationally easy to compute. Can methods derived from modular combination with shared information outperform these more efficient methods? If the shared information-based methods are truly distinct, can the large quantity of measures be leveraged for performance gains through ensemble integration techniques?

This work presents extensive and novel research on the under-studied effects of shared information in semantic calculations in the gene ontology through robust evaluations of their performance on traditional and real-world tasks. As no method can determine *true* semantic similarity between genes [13], various methods for evaluation have been put forward to evaluate the performance of semantic similarity methods. Commonly used evaluations include correlation with sequence similarity [11], [19], correlation with domain set similarity [13], [20], [27], correlations with gene expression [4], [23], [28], clustering genes into known pathways [3], [5], [21], and prediction of protein–protein interactions [3], [6], [29]. Based on these past works, we have constructed a new suite of six benchmarks to evaluate semantic similarity that provides evaluations for a broad range of use cases. These benchmarks are provided as lists of protein pairs and scores to facilitate use by other researchers. It is known that the GO annotations are incomplete and can suffer from bias and noise [30]. To address these issues, our evaluations use bootstrapping to provide robust statistical performance comparisons for each measure. Due to the large number of measures and the number of evaluations needed, no current tools addressed our simultaneous needs of speed and modularity. To achieve these goals, we have developed a new set of efficient, modular tools for working with GO graphs in C++, called the GO Graph Tool Kit (GGTK). With the aim of facilitating further research in the community, we provide an easy-to-use Python package that wraps the functionality of GGTK and release all GGTK code under the permissive BOOST License. GGTK will remain an ongoing open source project available at github.com/paulbible/ggtk.

## 2 Methods

### 2.1 Calculating information content and shared information

Understanding the structure of the GO DAG provides insight into how semantic similarity is derived at the term level. In GO graphs, each vertex or node represents a term or concept and each edge presents a relationship such as *is*_*a* or *part*_*of* [1]. A root node in GO represents the most general concept (e.g. *biological*_*process*), and all other concepts are considered descendants of this term. Information theoretic semantic measures rely on assigning a value of probability to a term. Lord et al. [11] proposed defining a term's probability as the number of times it occurs in a corpus of annotations divided by the number of occurrences for all terms. A term occurs if it or *any of its descendants* appear in the corpus. Eq. (1) shows the definition of probability where *o*_*t*_ is the number of occurrences for a term *t*. The information content (IC) follows, in Eq. (2), as the negative log of probability. As *o*_*t*_ includes all appearances of child terms, *P*(*root*) = 1 and any non-root term *t* satisfies *P*(*t*) < *P*(*root*). These definitions ensure IC is monotonically increasing toward more specific terms.

(1) $$P(t)=\frac{o_{t}}{o_{root}}$$

(2) IC(t)=−log(P(t))

Using the above definitions Lord et al. used the IC calculation in adapting Resnik [16], Lin [17], and Jiang-Conrath [18] to GO. The original shared information approach calculates the set of common ancestors (CA) between two ontology terms (Eq. (3)), and finds the most informative common ancestor (MICA) or equivalently, after Resnik [16], the probability of the minimum subsumer (Eq. (4)). The shared information of these traditional term similarity measures is defined in Eq. (5).

(3) $$CA(t_{1},t_{2})=Ancestors(t_{1})\cap Ancestors(t_{2})$$

(4) $$P_{ms}(t_{1},t_{2})=\underset{t\in CA(t_{1},t_{2})}{\min}\{P(t)\}$$

(5) $$\begin{array}{ll} SI_{MICA}(t_{1},t_{2}) & =IC\left(MICA(t_{1},t_{2})\right)\\ & =-\log(P_{ms}(t_{1},t_{2})) \end{array}$$

Using the above definitions, Resnik, Lin, and Jiang-Conrath semantic similarity (SS) can be re-written equivalently in terms of shared information (Eqs. (6), (7), and (8) respectively).

(6) $$SS_{Resnik}(t_{1},t_{2})=SI(t_{1},t_{2})$$

(7) $$SS_{Lin}(t_{1},t_{2})=\frac{2*SI(t_{1},t_{2})}{IC(t_{1})+IC(t_{2})}$$

(8) $$SS_{JC}(t_{1},t_{2})=1-(IC(t_{1})+IC(t_{2})-2*SI(t_{1},t_{2}))$$

### 2.2 Implementation considerations affecting time complexity

Before describing the different shared information algorithms and their time complexity, we will explain the implementation of GO used within the Go Graph Tool Kit (GGTK). Varying reports on the time complexity of semantic similarity algorithms have been put forward due to implicit assumptions about the representation of the GO DAG and associated annotations. Here we clarify the graph representation and its implementation. GGTK reads the ontology files (go-basic.obo) provided by geneontology.org [1], and stores the graph structure for the three disjoint ontologies (BP, MF, CC) in memory as adjacency lists. Let *n* be the number of terms in the ontology. Probability and information content are calculated from annotations taken from UniProt-GOA [31]. GGTK reads GO annotations from file, indexes annotations by gene, and provides gene-to-annotation look-ups in *O*(1) time. Calculation of IC is performed after Lord et al. [11] and is accomplished in *O*(*n*) time. Specifically, a depth first traversal visits every node in the GO graph. The number of annotation occurrences is calculated for each term starting with leaf nodes, moving to more general terms in the graph, and finishing with the root node. The number of cumulative occurrences for the root node is used to calculate term probability and IC after Eqs. (1) and (2) respectively. After the initial calculation, a function that maps a term to its IC is available as a map in which lookups are performed in *O*(1) time. In this work, IC was calculated using human annotations (GOA Dec. 2016) including electronically inferred annotations and considering only *is*_*a* and *part*_*of* relationships.

**Fig. 1:**
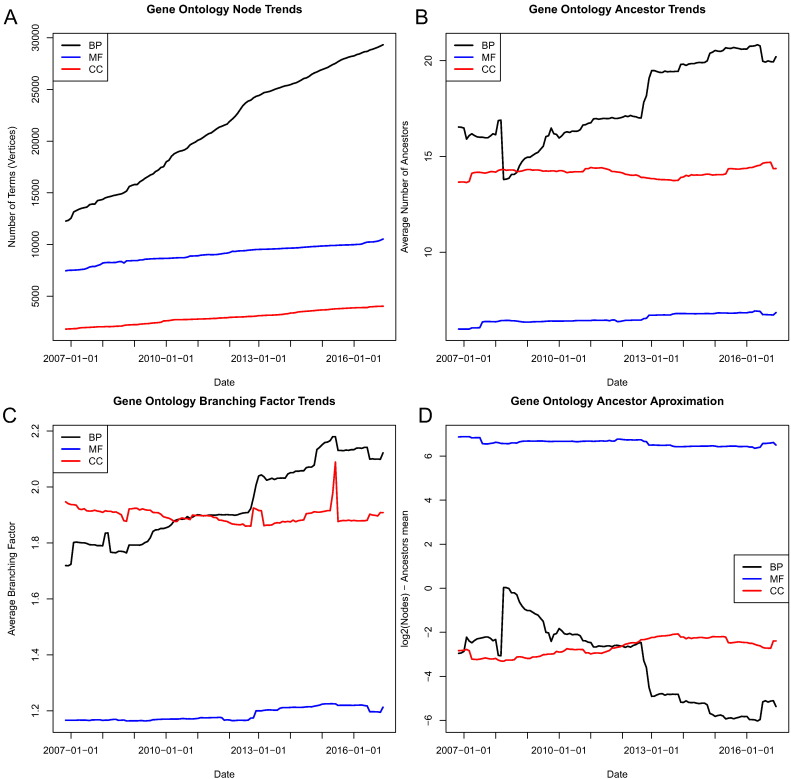
Changes in GO graph structure (using *is*_*a* and *part*_*of* relationships) over time lead to variations in (A) number of nodes, (B) mean ancestor number, and (C) the mean branching factor for each term. Panel D shows the log of the number of terms minus the mean ancestor number.

The semantic similarity methods under study in this work all rely on the calculation of the set of common ancestors shared between two concepts. The DAG structure of GO complicates estimates of the size of this set with respect to the number of terms, *n*. For a tree based ontology, the size of the common ancestor sets is bounded by *O*(log *n*), but this complexity is not guaranteed for general DAGs. Using GGTK we compiled historical data on the average branching factor and number of ancestors of each term from 2006 to 2016. Fig. 1 shows summary data for the three ontologies BP, MF, and CC (considering only *is*_*a* and *part*_*of* relationships) as the graph topology evolved over 10 years. The number of nodes, mean branching factor (node degree), and mean ancestor number are growing over time. Fig. 1D shows that log *n* is an under estimate in the case of BP and CC but over estimates the average number of ancestors in MF. After Couto et al. [20], we will refer to the number of common ancestors as *k* in our complexity analysis where |*CA*| = *O*(*k*). Based on our empirical analysis of the GO graph over the past 10 years, it appears that *O*(*k*) ≈ *O*(log *n*) for current and past graphs; however, this relation may not hold in the future.

### 2.3 Shared information algorithms and their time complexity

Determining the shared information between two ontology terms is an area of ongoing research. In this work we address the effects of five unique shared information algorithms on their derived gene functional similarity measures. A thorough analysis of these methods must address their time complexity as well as qualitative features. In this section, we provide a brief description of the shared information algorithms analyzed in this work, describe their features, and address their time complexity. A more detailed description of the algorithms can be found in Appendix A. The source code for all methods is available at github.com/paulbible/ggtk. The following complexity bounds refer to the calculation of single-term-to-single-term shared information.

#### 2.3.1 Common ancestor shared information

For purposes of evaluation, we developed a naive baseline algorithm called common ancestor shared information (CASI). CASI simply calculates the set of common ancestors and returns the mean IC as the shared information. This naive baseline serves as a useful tool for evaluating other shared information algorithms. The time complexity of CASI is *O*(*k*). Calculating the common ancestor set is performed in *O*(*k*) and using GGTK's *O*(1) IC map allows the mean to be calculated in *O*(*k*).

#### 2.3.2 Most informative common ancestor shared information

The most informative common ancestor (MICA) shared information remains the most common shared information measure as it forms the basis of traditional term similarity methods. (Eq. (5), above). MICA shared information is equivalent to Resnik term similarity [11], [16], and is defined as the IC of the MICA. Due to its simplicity, it is one of the most widely used shared information measures and forms the foundation for many more sophisticated semantic algorithms. The time complexity of MICA is *O*(*k*). Construction of the common ancestor set takes *O*(*k*) and finding the term with maximum IC in the set is also *O*(*k*).

#### 2.3.3 Couto et al. 2007, GraSM

Couto et al. devised GraSM [20] as an alternative to MICA. They observed that different paths in the ontology represent different interpretations of concepts. They reasoned that ancestors with multiple interpretations, designated disjunctive common ancestors (DCA), should factor into the shared information. Calculation of the DCA involves determining if a common ancestor has unique paths to each of the two terms that are separate from the other ancestors under consideration. For each common ancestor, *t*_*a*_, paths are counted to determine if *t*_*a*_ is disjoint from another ancestor, say *t*_*b*_. If the number paths from *t*_*a*_ to the two input terms under consideration is greater than or equal to the number of paths from *t*_*b*_ to the input terms, *t*_*a*_ must be disjoint and represent some unique interpretation of the shared information by virtue of having a unique path to the terms that does not pass though *t*_*b*_. This process is repeated for all pairs of common ancestors. A detailed description of the GraSM algorithm is provided in Appendix A.3. Couto et al. reported the GraSM time complexity as *O*(*k*^2^), but their implementation uses a pre-computed path number map. The term-to-term path number map can be calculated for a one-time cost of *O*(*n*^2^), where *n* is number of terms in the ontology. The parallelized version of this calculation can be performed by n separate topological sorts each taking *O*(*n*) time. This ‘one-time’ cost could be quite large as *n* ≫ *k* and would need to be recalculated anytime other relationship edges were considered. To calculate GraSM shared information in real-time requires *O*(*k*^3^) operations. This result follows from the *O*(*k*^2^) calculations required to constructing the DCA and an added *O*(*k*) cost for path counting at each step leading to a runtime that is cubic in the number of common ancestors.

#### 2.3.4 Adjusted GraSM

Close study of the behavior of GraSM on real-world datasets leads to some unexpected results. In many cases, the DCA comprises a large proportion of the common ancestor set including the root node. An illustration of this behavior is provided in Supplemental Information Trace 1. GraSM is calculated by averaging the IC of DCA terms and may decrease as more shallow terms are included. In determining the membership of the DCA set, GraSM uses a *greater than or equal to* comparison on the number of paths. By changing the *greater than or equal to* (≥) to a *strictly greater than* ( >) in the path number comparison, the algorithm's behavior changes and fewer terms are included in the DCA set. This modified algorithm is referred to as adjusted GraSM (A-GraSM). See Supplemental Information Trace 1 and 2 for further details on A-GraSM. The time complexity of the adjusted algorithm is the same as GraSM, *O*(*k*^3^).

#### 2.3.5 Semantic frontier

Zhang and Lai introduced the exclusively inherited common ancestors set as an alternative to the DCA [21] that calculated a subset of the DCA in linear time. Our group developed an efficient implementation of the algorithm by Zhang and Lai, called the semantic frontier (SF) algorithm. A simple analogy can help to explain the exclusively inherited common ancestor set. The common ancestor set can be considered a region of the GO graph. Terms that form the *semantic frontier* lie on the frontier of the territory formed by the common ancestors. Specifically, terms in the semantic frontier set are common ancestor terms that have an incoming edge leading from one of the input terms not shared by paths leading from the other. The average IC of this set is returned as the shared information. The SF implementation offers a reduced search space over the algorithm proposed by Zhang and Lai. The SF algorithm is based on the concept of a breadth first search (BFS) visitor. A BFS visitor performs actions when certain events in a BFS occur. A BFS is performed for each of the two input terms and the SF set is calculated by examining the visited edges entering the common ancestor set. A detailed description of the SF algorithm is available in Appendix A.5.

The time complexity of the SF algorithm is *O*(*k*). SF calculates the common ancestor set in *O*(*k*), each BFS visitor operates in *O*(*k*), and checking for frontier edges takes *O*(*k*) time. Zhang and Lai report the complexity of their algorithm as *O*(*n*log *n*) [21]. The greater complexity reported by Zhang and Lai may be the result of less efficient ancestor or IC access which GGTK provides in *O*(*k*) and *O*(1) time respectively. Based on the increase in GO graph size in past 10 years, our early exit and reduced search space features may yield further performance gains in the future.

### 2.4 From shared information to term similarity

Methods for calculating term similarity can be constructed from shared information calculations through substitution into Resnik, Lin, and Jiang-Conrath (see Eqs. (6), (7), and (8)). In order to construct term similarity calculators using these method, GGTK provides a shared information interface that allows the modular combination of shared information and term similarity algorithms. This combination allows multiple distinct term similarity measures to be constructed. The interface promotes extensibility allowing other researchers to construct shared information algorithms and immediately combine them with existing term similarity measures.

#### 2.4.1 Gene similarity from all-pairs of terms aggregation

The calculation of gene functional similarity relies on methods that aggregate the all-pairs term similarity between gene annotations [6]. GGTK provides *max*, *average*, and *best-match average* (BMA) gene similarity aggregators. Research by Pesquita et al. [19] and others has suggested that BMA outperforms other methods of aggregation. Based on initial experiments using the Collaborative Evaluation of Semantic Similarity Measures (CESSM) online tool, we arrived at the same conclusion. We evaluated the gene similarity benchmarks using BMA, but other aggregation methods may be useful in other contexts.

The number of gene-to-gene semantic similarity measures (*N*_*GSS*_) explodes in a combinatorial fashion as described in Eq. (9) where |*si*| is the number of shared information methods, |*ss*| the number of semantic term similarity methods, |*a*| the aggregation methods, and the final term, 3, represents the distinct ontologies, BP, MF, and CC. As new methods are devised to calculate term similarity, shared information, and aggregation of term similarity, the number of gene similarity measures grows combinatorially. This increasing makes evaluation a challenge, but offers a rich set of measures from which to choose. We explore whether these measures are significantly distinct in terms of performance and if gains can be achieved by using multiple measures.

(9) $$N_{GSS}=|si|*|ss|*|a|*3$$

#### 2.4.2 Jaccard-based gene similarity measures

Some gene similarity methods act on sets of terms without the need to explicitly calculate term-to-term similarity or employ aggregation of term similarity. To contrast the performance of shared information methods with other established approaches, we consider alternative gene similarity methods reported by the literature to perform well. Many of these approaches are variations of the Jaccard index [32] for set similarity. The Jaccard index calculates the ratio of the intersection to the union of sets. GGTK provides four Jaccard-based gene similarity measures. Gentleman [25] introduced a Jaccard-based gene similarity measure that calculates the ratio of shared ancestors between two terms to the union of each terms' ancestors. Let $A_{t}$ be the set of all ancestors of term *t* (including *t*). The set $A_{t}$ can be thought of as the induced subgraph of a term *t* in the ontology DAG. Eq. (10) describes the measure called SimUI, a similarity based on the union and intersection of term ancestors.

(10) $$SimUI(t_{1},t_{2})=\frac{\left|A_{t_{1}}\cap A_{t_{2}}\right|}{\left|A_{t_{1}}\cup A_{t_{2}}\right|}$$

Pesquita et al. [19] developed an information content weighted version of SimUI called SimGIC, a graph-based information content similarity. SimGIC is defined in Eq. (11).

(11) $$SimGIC(t_{1},t_{2})=\frac{\sum_{t\in A_{t_{1}}\cap A_{t_{2}}}IC(t)}{\sum_{t\in A_{t_{1}}\cup A_{t_{2}}}IC(t)}$$

Studies by Mazandu et al. [26] introduced two modified methods similar to SimGIC. These methods are called by those authors, SimDIC and SimUIC after Dice and *universal* indexes. These measures are defined in Eqs. (12) and (13).

(12) $$SimDIC(t_{1},t_{2})=\frac{2*\sum_{t\in A_{t_{1}}\cap A_{t_{2}}}IC(t)}{\sum_{t\in A_{t_{1}}}IC(t)+\sum_{t\in A_{t_{2}}}IC(t)}$$

(13) $$SimUIC(t_{1},t_{2})=\frac{\sum_{t\in A_{t_{1}}\cap A_{t_{2}}}IC(t)}{\max\left\{\sum_{t\in A_{t_{1}}}IC(t),\sum_{t\in A_{t_{2}}}IC(t)\right\}}$$

As these methods operate on the gene-level term sets, they avoid the all-pairs-of-terms calculation and need no aggregation step making these Jaccard-based methods more efficient than the shared information methods. The linear time shared information algorithms showed acceptable speed for all the practical applications of this work despite being necessarily slower than these Jaccard-based methods. For this reason, their execution time was not measured.

#### 2.4.3 Decoupling term similarity calculation from gene similarity

As some shared information methods are computationally inefficient, we wanted to decouple the calculation of shared information from the calculation of gene similarity. To achieve efficient calculation of gene similarity, GGTK provides capabilities to generate and import pre-computed term similarity matrices. By pre-computing term similarity, term similarity algorithms can operate on sets of terms and access the similarity of a pair of terms in *O*(1) time. Although the memory cost would appear to be *O*(*n*^2^) in the worst case, an optimization mitigates this cost by calculating only similarity between terms that appear in a given corpus (rather than all terms in the ontology). The separation of terms in each of the disjoint ontologies (BP, MF, CC) provides further space savings. If no gene in the corpus has a particular function annotation there is no need to calculate its similarity to other terms. These optimizations greatly reduce the memory cost and allow for efficient calculation of the gene-to-gene functional similarity.

#### 2.4.4 Measuring execution speed of shared information algorithms

The calculation of term similarity matrices also serves as a benchmark for measuring each algorithms' execution speed. In order to quantify the execution speed of the algorithms in each complexity class, the wall-clock time of each matrix calculation was recorded for each shared information algorithm using the Linux *time* command. In calculating the matrix, only those terms with annotations in the human corpus need to be analyzed. Using this optimization, the term similarity matrices were calculated processing 11,394 annotated terms for BP, 4149 for MF, and 1546 terms for CC. Taking advantage of symmetry (*Sim*(*A*,*B*) = *Sim*(*B*,*A*)), the resulting number of calculations equals the number of elements in the upper triangular portion of the term similarity matrix $\left(\frac{n(n-1)}{2}\right)$. Where the all-pairs-of-terms similarity is calculated, 64,905,921 pairs were processed for the BP, 8,605,026 for MF, and 1,194,285 for CC. The resulting wall-clock time for each of these calculations is reported as the execution time for each method.

### 2.5 Semantic similarity performance evaluations

Various performance evaluations have been put forward starting with Lord et al. [11] who measured the Pearson correlation between gene-to-gene semantic similarity scores and sequence similarity. Foundational approaches accepted in the literature measure the correlation between gene-to-gene semantic similarity and sequence, domain set, or expression profile similarity [14]. Perhaps more useful evaluations measure the predictive power of semantic similarity to discover protein–protein interactions [6], [29] or to correctly cluster genes belonging to known pathways [21]. Machine learning evaluations such as these have more applications in the field.

Few studies have addressed the inherent uncertainty in both GO graph structure and the limited depth and breadth of gene annotations [29]. The machine learning community has long used bootstrapping to improve generalization and to overcome issues of noise [33]. To address the issue of uncertainty, we employ robust bootstrapping approaches that provide generalized measures of performance as well as performance distributions that allow direct statistical comparisons between similarity methods. In the following sections we describe the specific performance evaluations and datasets used to compare a gene similarity method with respect to the shared information algorithms. In this work we evaluate five shared information algorithms using three term similarity measures across the three ontologies of GO resulting in 45 distinct gene similarity measures (5 shared information methods * 3 term similarity methods * 3 ontology types). In addition, these methods are compared to 12 gene similarity methods derived from the Jaccard-based methods (4 Jaccard-based methods * 3 ontology types). The performance of 57 measures in total has been analyzed.

#### 2.5.1 CESSM

The Collaborative Evaluation of Semantic Similarity Measures (CESSM) [34] is an online dataset and comparison tool used to evaluate gene similarity measures. Though CESSM has some issues [27], the community has accepted it as a useful but limited performance benchmark which can provide comparisons with 11 other measures. The CESSM annotation and test data was downloaded and used to evaluate each measure. The 45 shared information methods were submitted individually to the CESSM server and the results, once collected, were analyzed. CESSM is available at xldb.di.fc.ul.pt/tools/cessm/. CESSM was used to evaluate term aggregation techniques. BMA performed best on this benchmark. The aggregators *max* and *average* performed poorly and were not considered for subsequent analysis. This agrees with similar observations by Pesquita et al. [19] and others. This analysis motivated the choice of BMA and the results for each ontology type are available in Supplemental Information Figures S1–S3.

#### 2.5.2 Relative reciprocal BLAST score

The relative reciprocal BLAST score (RRBS) is a measure of sequence similarity developed by Pesquita et al. [19] derived from the BLAST alignment tool [35]. Eq. (14) gives the RRBS definition between two sequences *A* and *B* [19]. All-pairs BLAST was performed using an e-value cut off of 1e−4 after [19] with the human protein dataset. Pesquita et al. noted the relationship between shared information and RRBS is non-linear. For this reason, we evaluated the non-linear Spearman's rank correlation, or Spearman's *ρ*, between gene similarity values and the sequence alignment derived RRBS. The performance distribution of the Spearman correlation was calculated by taking 1000 bootstrap samples of size 100,000 from the set of all RRBS scores calculated.

(14) $$RRBS(A,B)=\frac{BLAST_{\text{bitscore}}(A,B)+BLAST_{\text{bitscore}}(B,A)}{BLAST_{\text{bitscore}}(A,A)+BLAST_{\text{bitscore}}(B,B)}$$

#### 2.5.3 Jaccard index set similarity of Pfam domains

The Jaccard index [32] measures the similarity between two sets as the size of their intersection divided by the size of their union. In general, the functional domains, rather than just sequence, dictate a protein's function. To this end, Pfam domains [36] have been used in various gene similarity measure evaluations [13], [20], [27]. Taking genes as sets of domains (*D*_*A*_ and *D*_*B*_), a domain similarity method is derived (Eq. (15)). On the human protein dataset, Pfam domains were predicted using HMMER3 [37] with an e-value threshold of 3*e*^ −6^. After removing gene isoforms, the all-pairs Jaccard Pfam similarity was calculated for the protein dataset. As above, performance distributions for each algorithm were generated by calculating the Spearman correlation between the gene similarity measure and Jaccard Pfam for 1000 bootstrap samples of size 100,000.

(15) $$Sim_{\text{Jaccard}}(D_{A},D_{B})=\frac{\left|D_{A}\cap D_{B}\right|}{\left|D_{A}\cup D_{B}\right|}$$

#### 2.5.4 TF–IDF cosine similarity of Pfam domains

Term frequency –inverse document frequency (TF–IDF) is a technique in information retrieval that weights terms by their relative specificity [38]. Similarly, domains that appear frequently in a diverse set of proteins may have little influence on the protein's function. Song et al. [39] previously applied TF–IDF to protein domains. Eq. (16) shows the weight for a particular domain *d* belonging to a protein, where *f*_*d*_ is the frequency of that domain in the protein, *N* is the total number of proteins in the corpus, and *n*_*d*_ is the number of proteins having the domain. From this weight measure, proteins are represented as a vectors of domain weights (*A* and *B*) and cosine similarity (Eq. (17)) represents the protein similarity. Performance distributions for this measure were calculated using Spearman correlation and bootstrapping as described above.

(16) $$w_{d}=f_{d}*\log\left(\frac{N}{n_{d}}\right)$$

(17) $$Sim_{TF-IDF}(A,B)=\frac{A\cdot B}{∥A∥∥B∥}$$

#### 2.5.5 Gene expression across 79 human tissues

Highly correlated genes are often functionally related. Studies in the literature [4], [23], [28] have evaluated performance by measuring gene-to-gene semantic similarity correlation with gene expression correlation. The gene similarity measures were evaluated in terms of their correlation with microarray gene expression across 79 human tissues (NCBI GEO accession GSE1133). Probes from the human U133A array were mapped to their Refseq identifiers which were then mapped to Uniprot identifiers. Genes without any GO annotations in GOA were removed. After filtering, the all-pairs correlation of 5688 genes was calculated resulting in 16,173,828 unique correlation pairs. Both Pearson and Spearman gene correlations were calculated. As in previous literature [6], the absolute value of the correlation was calculated between expression pairs to attempt to detect a relationship either negative or positive. The semantic methods were then evaluated for their Spearman correlation to either the absolute Pearson or Spearman expression correlation. From these correlation pairs, 100 bootstrap samples were taken for each algorithm. Each sample had a size equal to 15% of the total number of pairs ( >2.4 million).

#### 2.5.6 Reactome pathway analysis

Uncovering pathway relationships between genes constitutes an important use case for semantic similarity algorithms that has been studied in the literature [3], [5], [21]. Using Reactome [40], we tested the performance of gene similarity methods in terms of their ability to partition sets of genes into known pathways by clustering. The variation of information (VI) criterion [41] was used to determine the agreement between known Reactome pathways and partitions of genes derived from hierarchical clustering using Ward's method [42] and gene-to-gene semantic similarity based distances (1 - similarity). Specifically, 100 datasets were generated from Reactome by randomly selecting 10 human pathways having between 10 and 150 proteins. These datasets were further processed replacing any overlapping pathways with non-overlapping pathways to remove any ambiguous assignments. For each gene similarity method, distance was calculated and the proteins were clustered into 10 groups. VI was calculated between the gene similarity based clustering and the known pathway assignments from Reactome. The 100 separate datasets were used to construct performance distributions.

#### 2.5.7 Protein–protein interaction prediction

Several works in the literature [3], [6], [29] have used protein–protein interaction prediction to evaluate semantic similarity measures. Following previous methods, positive and negative datasets were generated from the Interologous Interaction Database (I2D) [43], and the semantic similarity measures were evaluated by their ability to distinguish interacting from non-interacting protein pairs. The I2D public version 2.3 was downloaded from http://ophid.utoronto.ca/ and used as the positive set of interacting proteins (228,847 human interactions). A negative dataset of equal size was generated after Guo et al. [3] by randomly choosing protein pairs resulting in a balanced dataset of 457,694 interactions. For each gene similarity method, 100 bootstrap samples of a size equal to 15% of the original dataset were used to calculate receiver operator characteristic (ROC) curves using the ROCR package [44] of the R programming language. A ROC curve plots the true positive rate (TPR), the ratio of true predictions to all predictions made, against the false positive rate (FPR), the ratio of false predictions to all prediction, across a range of different thresholds. For the gene-to-gene semantic similarity measures, the ROC curve would be equivalent to sorting all the values of semantic similarity and counting the number of true and false predictions below each unique value for plotting. From these 100 samples, a performance distribution of the area under the ROC curve (AUC) values was used to statistically compare the performance of each semantic similarity measure. A classifier must have an AUC above 50% to be considered better than random guessing.

### 2.6 Isolating the effect of shared information from other factors

The choice of shared information, term similarity algorithm, and ontology type specify the gene-to-gene semantic similarity measure. To determine the effects of shared information on performance in the previous evaluations it is important to isolate the effects of other factors that influence the results.

#### 2.6.1 Regression analysis of mean performance

Linear regression analysis on the bootstrapped mean performance values is used to examine the effects of shared information type, term similarity algorithm, and gene ontology type. A design matrix was created with each row representing a separate gene-to-gene semantic similarity method constructed through combining the different factors under study. Each column of the design matrix represents the different factors as categorical variables, with the ontology variable taking a value in {*BP*, *MF*, *CC*}, the term similarity variable taking a value in {*Resnik*, *Lin*, *JC*}, and the shared information variable taking a value in {*casi*, *mica*, *grasm*, *agrasm*, *sf*}. Using this design matrix, linear models were trained for each performance evaluation using the mean bootstrapped performance as the response variable. The models where creating using the *lm* function of the R programming language. The influence of each factor is reported as the negative log of the regression p-value taken from the analysis of variance of each fitted model using the *anova* function of the R programming language. Separate models were fitted for each of the six performance benchmarks and the relative influence of each factor is reported for all evaluations.

#### 2.6.2 Performance ranking and statistical ties

Within each choice of term similarity and ontology type, the shared information algorithms were ranked based on statistical tests of their performance. For a specific choice of term similarity and ontology type, the shared information methods were sorted based on their mean performance and ranked using a statistical method operating on their performance distributions. A statistical tie in performance between two methods is determined if a t-test of the methods' performance distributions fails to reject the null hypothesis of equal means (p-value  > 0.05, Welch's two sample t-test). The ranks are exhausted meaning that if two methods are tied for first place, method 1 and 2 receive the rank of 1, but the next best performer receives the rank of 3 (rather than 2). The overall ranks of all shared information methods are reported for every combination of term similarity and ontology type. The average shared information algorithm rank is reported for each ontology type and term similarity method.

### 2.7 Ensemble classifiers and cross-validation

With such a large number of gene similarity approaches, could they be combined through ensemble methods to improve performance on machine learning tasks? To answer this question, we applied majority voting to the task of protein-protein interaction prediction. A majority voting classifier makes a prediction based on the votes of a panel of other classifiers [45]. Although the area under the ROC curve provides a good estimate of a classifier's behavior across a range of thresholds, in practice, a single threshold must be chosen for a particular task. A process that maximized the F-score over a set of training data selected the threshold for an individual gene-to-gene semantic similarity method to be used as a classifier. The F-score represents the trade off between making true predictions and limiting false predictions. It is the harmonic mean of precision and recall and is defined in Eqs. (18), (19), and (20). These values were calculated using the ROCR [44] package in R. This training process was repeated for every individual method. From these individual classifiers, voting classifiers were constructed that predicted an interaction only if a majority of its constituent classifiers also predicted an interaction. Three ontology specific voting classifier were designed to include only methods in BP, MF, or CC categories. These classifiers helped to determine if any advantage could be derived from ensemble classifiers limited to a single GO type. A final voting predictor was constructed which consisted of all individual methods.

(18) $$\text{precision}=\frac{\text{true positives}}{\text{true positives}+\text{false positives}}$$

(19) $$\text{recall}=\frac{\text{true positives}}{\text{true positives}+\text{false negatives}}$$

(20) $$\text{F-score}=2*\frac{\text{precision}*\text{recall}}{\text{precision}+\text{recall}}$$

Ten-fold cross-validation was employed to test the performance of all individual classifiers as well as the four ensemble classifiers. Using the protein interaction dataset from Section 2.5.7, ten random partitions, or folds, were constructed each containing approximately 10% of the original 457,694 interactions. The classifiers were trained using nine folds and tested for the percent of misclassified instances (error rate) on the remaining fold. This process was repeated ten times. The classification error is reported for all 57 methods including the four voting classifiers.

## 3 Results

### 3.1 Execution time performance of the shared information algorithms

**Fig. 2:**
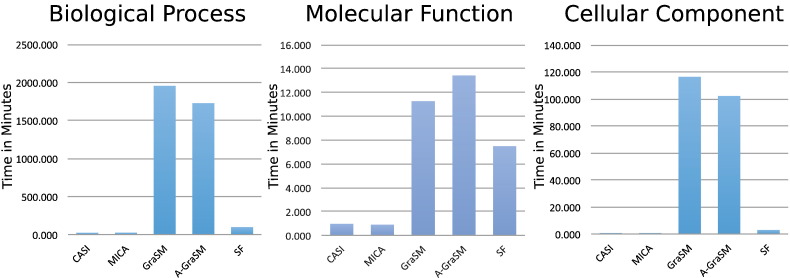
The execution times for the all-pairs term similarity for Resnik term similarity show that GraSM and A-GraSM methods are slower than other shared information methods.

The all-pairs term similarity matrix for annotated terms in BP, MF, and CC was calculated for each shared information method and the execution times of these calculations were recorded. The wall-clock execution times for these calculations showed, as expected, that GraSM and A-GraSM methods are orders of magnitude slower than the other shared information methods. Fig. 2 shows the execution times of all shared information methods combined with Resnik term similarity. The other term similarity measures showed comparable execution times. The complete list of execution times is available in Table S1. Both GraSM and A-GraSM have *O*(*k*^3^) time complexity, and their execution times dwarf the runtimes of the other algorithms. The execution time of the SF algorithm is greater than CASI or MICA. Both CASI and MICA proved to be the most efficient algorithms in terms of execution speed. The term similarity matrices for BP, MF, and CC required 64.91 million, 8.60 million, and 1.19 million term pair calculations respectively. Interestingly, despite requiring over 8 million more calculations, the MF processes completed faster than the CC processes. These results suggest that the topology of the GO graph plays an important role in determining the execution speed of semantic algorithms and that functions of the raw number of terms in an ontology may not accurately reflect their complexity. Fig. 1 supports this conclusion since the branching factor and average number of ancestors of the MF ontology is less than that of the CC ontology despite MF having more terms. The Jaccard-based methods do not require calculating term-to-term similarity so this evaluation does not apply. The Jaccard-based term-set level measures are known to be more efficient. These findings illustrate that the increased time complexity of the GraSM methods could be computationally prohibitive in some situations, and the problem may worsen as GO graph complexity grows.

### 3.2 RRBS: sequence similarity

**Fig. 3:**
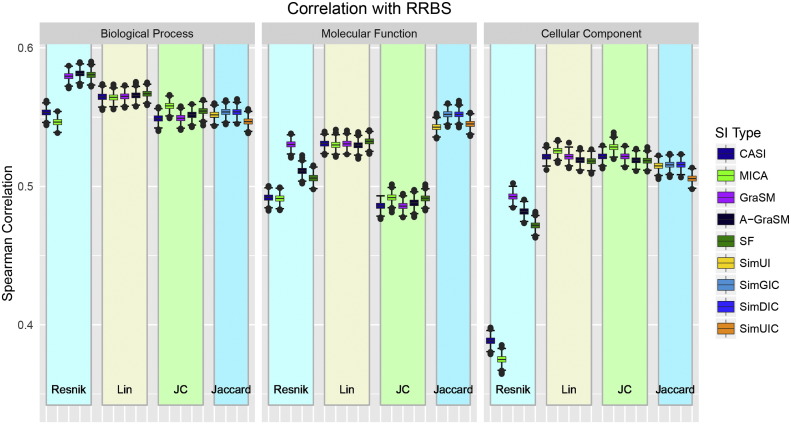
Performance distributions for the BLAST-based RRBS benchmark for all 57 measures organized by term similarity algorithm and ontology type.

Using the 519,892 protein pairs that passed selection, Spearman's *ρ* correlation between RRBS and each of the 45 shared information-based and 12 Jaccard-based gene similarity measures was evaluated by the bootstrapping method described in the Section 2.5.2. The correlation distributions of all measures are shown in Fig. 3. Supplemental Information Table S2 provides the mean performance and standard deviations for all measures. The measures are organized by term similarity type and ontology type. In general, BP methods perform best in terms of RRBS correlation. For Lin and Jiang-Conrath methods no dramatic differences are observed between shared information algorithms. For the Resnik methods, CASI and MICA lag behind GraSM, A-GraSM, and SF across all ontology types. With the CC ontology, the CASI and MICA Resnik methods show dramatically lower correlation than all other methods. Despite poor and average performance in the CC and MF ontologies respectively, Resnik BP methods using GraSM, A-GraSM, and SF show better correlation with RRBS than any other methods. Of the MF methods, Jiang-Conrath term similarity shows the lowest RRBS correlation among the term similarity algorithms. The Jaccard-based methods perform best out of the MF methods. The correlation of the MF Jaccard methods roughly equals the performance of the BP Jaccard methods. In the BP ontology, the Jaccard methods showed similar performance to the Jiang-Conrath methods but were lower than Lin and the best Resnik methods. These results show that BP ontology methods most closely correlate with RRBS similarity. Consistent with the results of Pesquita et al. [19], SimGIC performed well among the MF methods. In that work the authors did not consider BP or CC based methods citing the works Lord et al. [11], [12] that report a loose correlation between gene semantic similarity and sequence similarity. Jain and Bader [4] analyzed the correlation between semantic methods and sequence similarity in all ontologies, and their results show the CC methods have a higher sequence correlation than MF or BP. From the results of Mazandu et al. [26], BP and CC methods appear to show better correlation with sequence than MF. As Pesquita et al. [19] demonstrated the relationship between semantic similarity and sequence similarity is not linear, making the Spearman correlation a more appropriate measure. The use of Spearman correlation over Pearson and using more recent annotation and GO graph data likely account for some of the differences with previous works.

Statistical comparisons between performance distributions examine the relative performance between the methods and establish a ranking. This approach to ranking is described in Section 2.6.2. Table 1 shows the rankings for all shared information methods. These data give insight into which shared information algorithms show statically significant differences in performance for each combination of term similarity and ontology type. SF shared information works best with BP and MF methods. CC methods work well with GraSM on the RRBS benchmark. GraSM and A-GraSM show good performance with Resnik. Lin based method showed CASI, GraSM, and SF as the best shared information methods. MICA shared information ranked first in all JC methods.

### 3.3 Jaccard index of Pfam domain sets

**Fig. 4:**
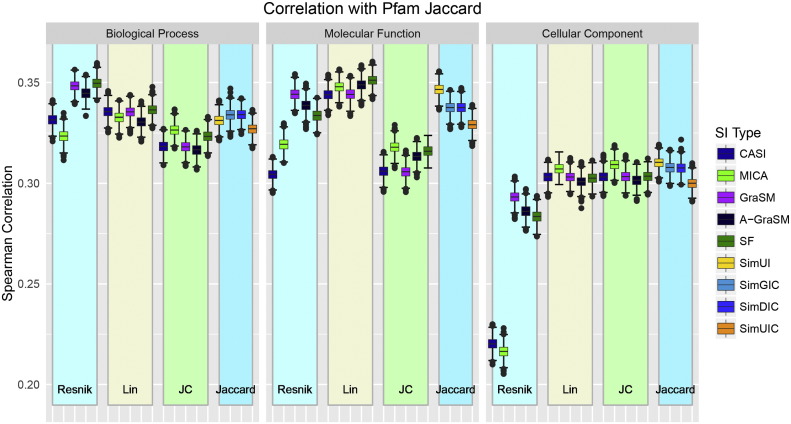
Performance distributions for the Pfam Jaccard benchmark for all 57 measures organized by term similarity algorithm and ontology type.

The all-pairs Pfam domain similarity was calculated with the Jaccard index resulting in 1,219,558 protein pairs. The performance distributions are shown in Fig. 4 depicting all 57 measures organized by term similarity type and ontology type. The complete listing of mean correlation performance and standard deviations can be found in Supplemental Information Table S3. As with the RRBS dataset, the shared information methods show smaller variability in performance compared to differences in ontology type and term similarity algorithm. The CASI and MICA methods combined with Resnik term similarity again perform poorly compared to the other methods, but these shared information method are comparable with GraSM, A-GraSM, and SF when combined with Lin and Jiang-Conrath. The CC ontology methods lag the performance of MF and BP in terms of correlation with Pfam Jaccard similarity. The Jaccard-based gene similarity methods perform best with MF annotations, with the best method being SimUI. These methods perform slightly better in MF than in BP. Overall MF Lin methods performed best, but BP Resnik methods combined with GraSM, A-GraSM, and SF outperformed most other methods. As the worst performing ontology group, the CC ontology may fail to encode detailed domain or structural information. Despite having a higher correlation than the CC ontology methods, the BP and MF correlations were lower than in the RRBS benchmark with the best methods only achieving a correlation near 0.35.

Table 2 shows the ranking of shared information methods for the Pfam Jaccard evaluations. Among the ontologies, SF performs best with BP and MF as in the RRBS evaluations. GraSM again shows the best performance for CC methods. Resnik, Lin and Jiang-Conrath method perform best with GraSM, SF, and MCIA respectively on the Pfam Jaccard dataset.

### 3.4 TF–IDF cosine similarity of Pfam domain sets

**Fig. 5:**
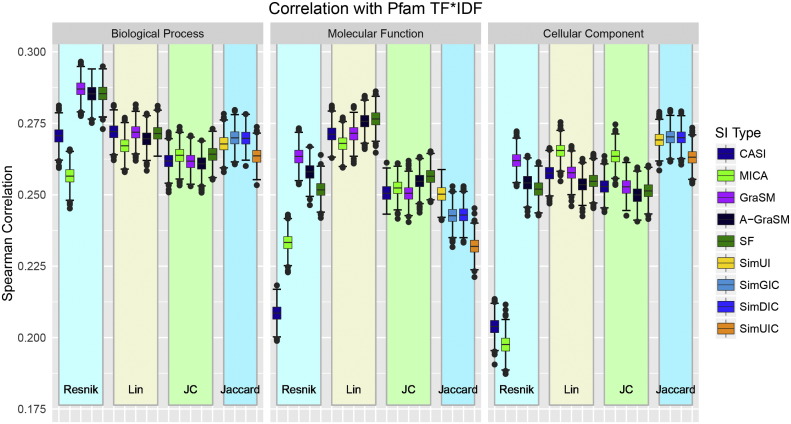
Performance distributions for the Pfam TF–IDF benchmark for all 57 measures organized by term similarity algorithm and ontology type.

To correct for uninformative domains, we applied TF–IDF to proteins represented as sets of Pfam domains. With 1,219,558 unique protein pairs, evaluations were conducted using the bootstrapping method. Fig. 5 shows the distribution of Spearman correlations against TF–IF domain similarity (mean and standard deviation data are available in Supplemental Information Table S4). Again, shared information methods show less variability with the exception of Resnik methods using CASI and MICA. CASI and MICA Resnik methods are dramatically low in the CC ontology and show the lowest correlations of all measures. BP Resnik methods using GraSM, A-GraSM, and SF outperform all other methods in terms of TF–IDF correlation. The Jaccard-based methods perform similarly in BP and CC ontology, but these methods are among the worst performing measures in MF. The CC Jaccard-based methods show greater correlation with TF–IDF than the other CC methods. The BP methods preform best on average in this evaluations. These novels findings suggest that TF–IDF can be successfully applied to protein domain sets to correct for nonspecific domains and potentially uncover relationships in both biological processes and molecular functions.

The performance rankings of the shared information methods in the TF–IDF benchmark are shown in Table 3. With BP and CC methods in the TF–IDF benchmark, GraSM shows the best average ranking. SF shared information works best with MF methods in this dataset. Resnik methods work best with GraSM which is ranked first in all Resnik evaluations. Lin methods show the best correlations using CASI and GraSM while Jiang-Conrath methods perform best with MICA and SF shared information.

### 3.5 Gene expression across 79 tissues

**Fig. 6:**
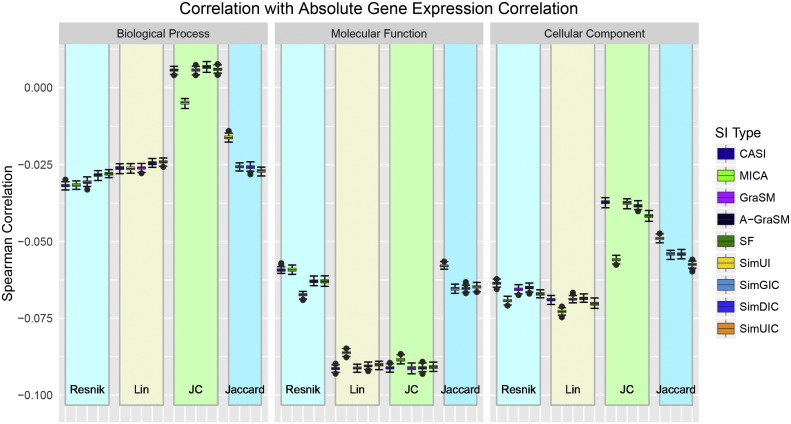
Performance distributions for absolute gene expression correlation (Pearson) against all 57 measures organized by term similarity algorithm and ontology type.

All 57 measures were evaluated based on their correlation with gene expression correlation. Fig. 6 shows the absolute gene expression correlation for all methods under study. The complete mean and standard deviation data are available in Supplementary Information Table S5 (Pearson gene correlation) and Table S6 (Spearman). BP Jiang-Conrath methods show the best performance at slightly greater than 0. All other methods show a negative correlation with expression correlation. These findings are consistent with the work of Xu et al. [6] which found that global gene expression correlates poorly with semantic similarity. Xu et al. observed a steady increase in semantic similarity as gene expression pairs are binned into sets of highly correlated gene sets. The same trend is confirmed in our data (Supplemental Figure S4). At such low levels of correlation, one might expect greater variability in performance due to a lack of any strong relationship. The data in Fig. 6 surprisingly shows tighter distributions than in other evaluations. The differences in performance between shared information types are minimal, with the exception of MICA.

The shared information rankings are provided in Table 4 for absolute gene expression. BP, MF, and CC methods work best with SF, MICA, and CASI shared information respectively for gene expression. CASI performs best with Resnik methods and A-GraSM with Lin methods. CASI, GraSM, and A-Grasm work well with Jiang-Conrath methods. The low and negative correlations associated with this benchmark makes the significance of this ranking less clear.

### 3.6 Reactome clustering

**Fig. 7:**
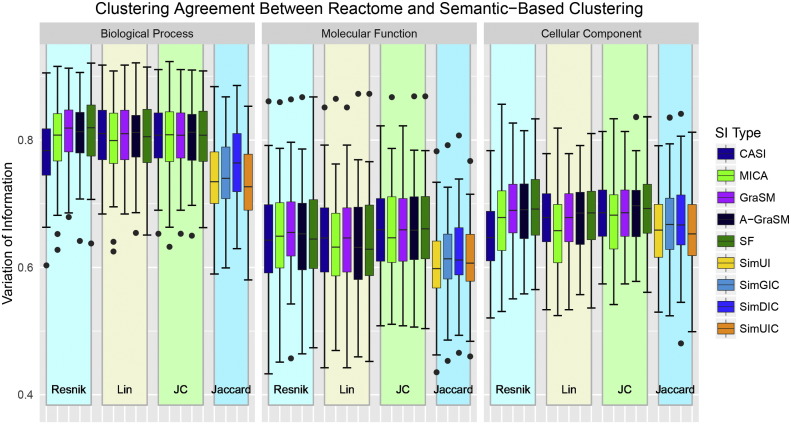
Performance distributions for Reactome clustering compared to the 57 gene similarity semantic measures organized by term similarity algorithm and ontology type.

Using 100 randomly generated pathway datasets (see Section 2.5.6), the mean VI similarity (1 - normalized VI distance) was used to compare the closeness of semantic gene similarity derived clusters to their known Reactome assignments. Fig. 7 shows the VI similarity distribution for all 57 methods tested. The performance variability on this benchmark is much higher than in other evaluations. The BP methods outperform those of MF and CC, but within each ontology type all shared information based methods are essentially equivalent regardless of term similarity type. The Jaccard-based method tend to under perform the other methods. As the Reactome pathways were chosen randomly, the high variability in performance is expected. The success of BP methods confirms expectations since the BP ontology captures functional information most closely associated with biological pathways. The CC based methods out-perform MF method on average for this benchmark. The complete results are available in Supplemental Information Table S7.

Table 5 gives the performance rankings of the shared information methods for the Reactome benchmark. As the Reactome results show high variability, most methods could not be determined to be statistically different from one another. All shared information methods within each term similarity group proved to be equivalent in the MF ontology in terms of the VI similarity to Reactome pathway clusters. In BP, the CASI method performs poorly with Resnik. In the CC ontology, CASI and MICA perform poorly with MICA particular ill suited to the Lin and Jiang-Conrath methods. Resnik methods show poor performance with CASI and MICA, while MICA Lin and Jiang-Conrath methods show poor performance reflecting issues in combination with the CC ontology.

### 3.7 Protein–protein interaction prediction

**Fig. 8:**
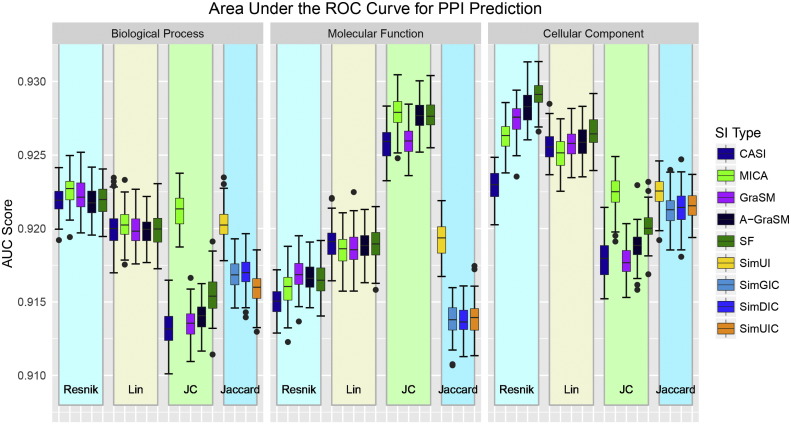
Performance distributions for protein–protein interaction prediction by area under the ROC curve for the 57 gene similarity semantic measures organized by term similarity algorithm and ontology type.

The predictive power of each gene similarity measure was evaluated on the human I2D interaction dataset. Performance distributions of the AUC (described in Section 2.5.7) appear in Fig. 8. Complete data for the mean and standard deviation of the AUC are available in Supplemental Information Table S8. This prediction-based evaluation showed heightened variability between shared information methods compared to other datasets, especially with the Jiang-Conrath methods. Within Jiang-Conrath term similarity, MICA shared information performed best. The shared information methods varied greatly within CC Reskin with the SF and A-GraSM versions giving the best AUC values of all methods. The Jaccard-based methods show the worst performance among MF methods and are superior to only the Jiang-Conrath methods in the BP and CC ontologies. BP and MF methods tend to under perform compared to CC methods with the exception of MF Jiang-Conrath. This result is at odds with older evaluations such as Guo et al. [3] that found BP methods are better predictors than CC methods. These differences are likely due to the smaller dataset used in their analysis as well as the changes in GO structure and annotations in recent years. Conflicting performance reports in the literature illustrate the need for transparent open source tools that can evaluate these methods on a level playing field using consistent ontologies and annotations.

The performance rankings for the protein–protein interaction prediction benchmark is shown in Table 6. Based on the distribution of the area under the ROC curve values, the shared information methods were statistically compared. MICA, A-GraSM, and SF shared information perform best for BP, MF, and CC ontologies respectively. GraSM and A-GraSM perform well for Resnik on this benchmark. CASI is the best performer for Lin term similarity, and MICA works best for JC across all the ontologies in these evaluations.

### 3.8 Analysis of factors affecting semantic similarity performance

**Fig. 9:**
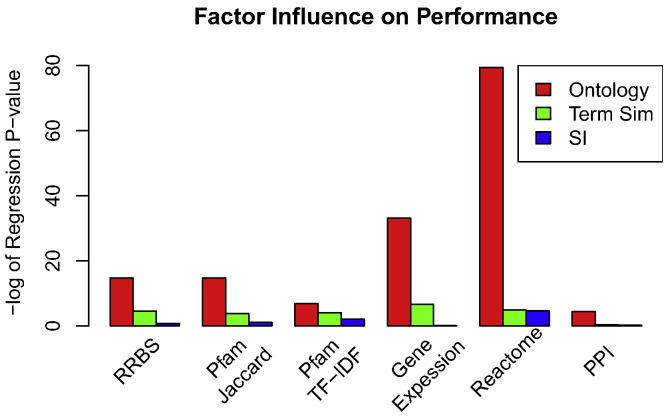
The relative influence of ontology type, term similarity method, and shared information type (SI) on the mean performance across six evaluations.

Using the six performance benchmarks described in the previous section, regression models were trained to assess the influence of ontology type, term similarity type, and shared information type on the mean performance of the shared information methods. A design matrix of categorical variables was constructed and used to create a linear model of mean performance (described in Section 2.6.1). Fig. 9 presents the influence of each factor on each benchmark as the negative log of the regression p-value. This data demonstrates that the choice of ontology contributes most to the variability in performance followed by the choice of term similarity, and shared information contributes the least. All evaluations tested uphold this trend. The Reactome benchmark shows the strongest influence by ontology followed by the gene expression evaluations. Ontology type exerts the smallest influence on TF–IDF and PPI benchmarks; however, this effect is still much greater than the other two factors. The effects of term similarity and shared information choice are small across all evaluations except PPI where the effects are negligible or zero. These results clearly show that the choice of ontology greatly effects the performance of gene similarity methods.

### 3.9 Leveraging semantic methods with majority voting

**Fig. 10:**
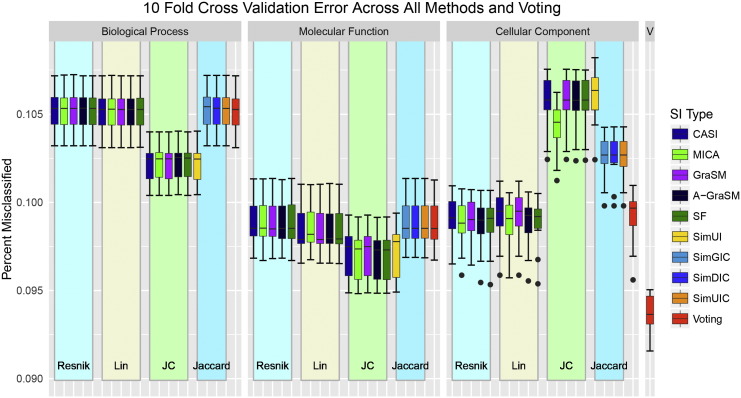
The percent of misclassified samples for each method under study, trained as classifiers, and four voting predictors evaluated by 10-fold cross-validation. The voting predictor for an ontology type is presented as the last classifier within that ontology (red), and the voting predictor utilizing all semantic methods is presented at the far right.

Using the protein–protein prediction dataset from the previous evaluations, we addressed the feasibility of combining semantic similarity methods to improve prediction. Using the 10-fold cross-validation method (described in Section 2.7), we analyzed the performance of all 57 measures in terms of their mean prediction error. For this evaluation, the percent of misclassified instances represents the classification error of each gene similarity semantic measure and voting classifier. Fig. 10 presents the cross-validation classification error for all methods organized by ontology type and term similarity method (lower is better). The ontology specific voting classifier performed equivalently with other methods within the ontology (the far right classifier within each ontology group in Fig. 10). Other single classifiers outperform the ontology specific voting predictor within each ontology. This result indicates that simple majority voting offers no advantage over the best methods within an ontology group; however, the combination of all semantic similarity methods substantially out performs even the best individual classifier. The failure of ontology specific voting predictors to confer any advantage for prediction suggests that variation among shared information or term similarity semantic methods alone cannot improve learning at least for this task. Analysis of the factors affecting semantic similarity performance of the previous section support this conclusion. The cross-validation evaluation show that BP methods tend to perform worse than all MF methods and worse than all but Jiang-Conrath CC methods. Interestingly, the MF methods perform best in cross-validation analysis while the CC methods tend to perform best in terms of the area under the ROC curve calculated through bootstrapping. These results indicate that the AUC metric may not totally capture the usefulness of a measure for classification.

## 4 Discussion

As semantic similarity measures have grown in popularity, a large number of measures have been developed making exhaustive performance assessment a growing challenge. IC based methods use measures of shared information to calculate term similarity and ultimately gene similarity. Recent works have devised alternative methods for calculating shared information. Through modular combination of shared information measures, term similarity calculations, and ontology choice, the number of semantic gene similarity measures explodes creating obvious challenges for performance evaluations. In a departure from previous works, we put forward robust methods to statistically compare semantic gene similarity measures in a manner that captures their generalized performance. We apply these methods to conduct a thorough investigation into the behavior of varying shared information measures. Given the large number of measure that can be created though modifications to the shared information, we considered the feasibility of leveraging these measures in ensemble classifiers for prediction gains. By isolating the effects of ontology type, we determined that the shared information algorithms themselves could not be combined to improve protein-protein interaction prediction. Methods across the distinct ontologies can be leveraged to improve prediction.

Xu et al. [6] combined information from across ontologies to improve prediction; however, they incorporated an artificial root node connecting BP, MF, and CC into a single graph. Their research found that BP methods offered the best performance on a yeast dataset. Later Jain and Bader [4] also showed BP to out perform MF and CC methods in interaction prediction in yeast based on area under the ROC curve analysis both with and without the inclusion of electronically inferred annotations. While our work focused on human protein–protein interactions, the results resemble the more recent work of Yang et al. [29] in yeast where they found CC to perform better than other methods. Based on ROC analysis, our results showed CC methods performing best. The work by Yang et al. cites Collins et al. [46] in identifying that much of the ROC curve represents classification thresholds that would be useless in practice for protein interaction prediction. This results from thresholds that admit far too many false positive predictions to be useful. This issue became evident in our analysis when many of the MF predictors showed strong performance based on cross-validation evaluation but less competitive performance when only the area under the ROC curve was considered.

The work by Mazandu et al. [26] more closely relates to this study as they used human interactions to assess the predictive power of semantic algorithms. The data set used by Mazandu et al. was comprised of a much smaller set of roughly 5000 curated interaction which were filtered to all contain BP and CC annotations. Citing their previous work [47], Mazandu et al. excluded MF based methods in their evaluation, and other work by Mazandu et al. [48] supported the exclusion of Jiang-Conrath methods. These caveats make a direct comparison to their work difficult. Mazandu et al. examined a large number of diverse measures which are out of score for this study of shared information methods. The shared methods among the two studies are the best match average (BMA) versions of Resnik (called RBMA, by Mazandu et al.), Lin (LBMA), SimUI, SimGIC (AGIC), SimDIC (ADIC), and SimUIC (AUIC). The area under the ROC curve reported Mazandu et al. for the CC base methods is 0.9999656 (RBMA), 0.4853167 (LBMA), 0.8483416 (SimUI), 0.9173889 (AGIC), 0.8486233 (ADIC), and 0.9654985 (AUIC). The values reported for the BP verions of these methods are 0.9995277 (RBMA), 0.6194642 (LBMA), 0.9582268 (SimUI), 0.9689432 (AGIC), 0.9514534 (ADIC), and 0.9654985 (AUIC). The range of these values is consistent with those found in this study for the area under the ROC curve benchmark. All the methods tested achieved high performance using the most up-to-date annotations and GO graphs. Inconsistent with the reports of this study is the disparity between Resnik and Lin methods within the same ontology. Based on our evaluations, we would expect these methods to have scored more closely in the evaluations of Mazandu et al.

Difficulty in comparing the results of semantic similarity analysis in GO is a known problem [14]. In an excellent review, Mazandu et al. [15] describe two key challenges known as the *dataset issue*, where different tools use different version of GO or annotation datasets, and the *scaling issue* that results from tools making different assumption regarding normalization methods and other minor considerations such as root membership in ancestor sets etc. GGTK is an attempt to provide correct, transparent, and modular implementations of semantic algorithms where the dataset issue can be tackled easily and the assumptions that could lead to the scaling issue are clearly stated. The benchmarks used in this work are provided as simple lists of protein pairs and scores to facilitate comparisons by other researchers (available at github.com/paulbible/ggtk). This work demonstrates that many conclusions from the literature still hold despite changes in the structure of the ontologies and increases in the number of annotations. In opposition to previous reports, we find that MF methods performed best in predicting protein interactions using cross-validation. We found that an ensemble of gene similarity semantic methods could out perform any single method in interaction prediction, but this boost in performance results mainly from the integration of distinct information gained through the separate ontologies of BP, MF, and CC. Furthermore, we have developed an efficient C++ toolkit with an easy-to-use Python interface, GGTK, that will not only allow other researchers to take advantage of these findings but also facilitate their own research into semantic methods.

## Figures and Tables

**Figure:**
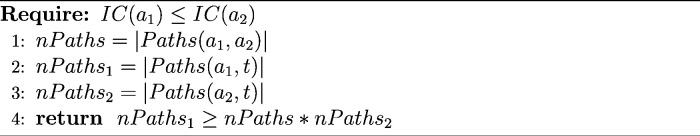


**Figure:**
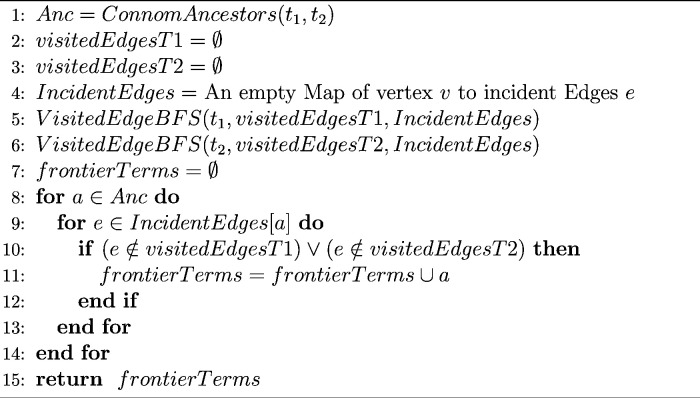


**Figure:**
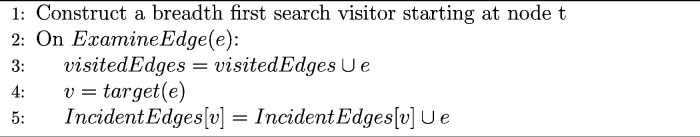


**Table 1:** Ranking of shared information for the BLAST-based RRBS benchmark against ontology type and term similarity type. Methods with greater correlation have lower rank. Bold font represents the best average rank for each category.

	Biological process				Molecular function				Cellular component				Mean by term similarity		
Method	Resnik	Lin	JC	Mean	Resnik	Lin	JC	Mean	Resnik	Lin	JC	Mean	Resnik	Lin	JC
CASI	4	3	5	4.00	4	2	4	3.33	4	2	2	2.67	4.00	**2.33**	3.67
MICA	5	5	1	3.67	5	4	1	3.33	5	1	1	2.33	5.00	3.33	**1.00**
GraSM	3	3	4	3.33	1	2	4	2.33	1	2	2	**1.67**	**1.67**	**2.33**	3.33
A-GraSM	1	2	3	2.00	2	5	3	3.33	2	4	4	3.33	**1.67**	3.67	3.33
SF	2	1	2	**1.67**	3	1	2	**2.00**	3	5	5	4.33	2.67	**2.33**	3.00

**Table 2:** Pfam Jaccard ranking of shared information against ontology type and term similarity type. Methods with greater correlation have lower rank. Bold font represents the best average rank for each category.

	Biological process				Molecular function				Cellular component				Mean by term similarity		
Method	Resnik	Lin	JC	Mean	Resnik	Lin	JC	Mean	Resnik	Lin	JC	Mean	Resnik	Lin	JC
CASI	4	2	3	3.00	5	4	4	4.33	4	2	4	3.33	4.33	2.67	3.67
MICA	5	4	1	3.33	4	3	1	2.67	5	1	1	2.33	4.67	2.67	**1.00**
GraSM	2	2	3	2.33	1	4	5	3.33	1	2	2	**1.67**	**1.33**	2.67	3.33
A-GraSM	3	5	5	4.33	2	2	3	2.33	2	5	5	4.00	2.33	4.00	4.33
SF	1	1	2	**1.33**	3	1	2	**2.00**	3	4	2	3.00	2.33	**2.00**	2.00

**Table 3:** Pfam TF–IDF ranking of shared information by term similarity type and ontology. Methods with greater correlation have lower rank. Bold font represents the best average rank for each category.

	Biological process				Molecular function				Cellular component				Mean by term similarity		
Method	Resnik	Lin	JC	Mean	Resnik	Lin	JC	Mean	Resnik	Lin	JC	Mean	Resnik	Lin	JC
CASI	4	1	3	2.67	5	3	4	4.00	4	2	2	2.67	4.33	**2.00**	3.00
MICA	5	5	2	4.00	4	5	3	4.00	5	1	1	2.33	4.67	3.67	**2.00**
GraSM	1	1	3	**1.67**	1	3	4	2.67	1	2	2	**1.67**	**1.00**	**2.00**	3.00
A-GraSM	2	4	5	3.67	2	2	2	2.00	2	5	5	4.00	2.00	3.67	4.00
SF	2	3	1	2.00	3	1	1	**1.67**	3	4	4	3.67	2.67	2.67	**2.00**

**Table 4:** Absolute gene correlation benchmark ranking for shared information methods organized by term similarity type and ontology. Methods with greater correlation have lower rank. Bold font represents the best average rank for each category.

	Biological process				Molecular function				Cellular component				Mean by term similarity		
Method	Resnik	Lin	JC	Mean	Resnik	Lin	JC	Mean	Resnik	Lin	JC	Mean	Resnik	Lin	JC
CASI	4	3	3	3.33	1	5	3	3.00	1	2	1	**1.33**	**2.00**	3.33	**2.33**
MICA	4	3	5	4.00	1	1	1	**1.00**	5	5	5	5.00	3.33	3.00	3.67
GraSM	3	3	3	3.00	5	4	3	4.00	3	2	1	2.00	3.67	3.00	**2.33**
A-GraSM	2	2	1	1.67	3	3	3	3.00	2	1	3	2.00	2.33	**2.00**	**2.33**
SF	1	1	2	**1.33**	3	2	2	2.33	4	4	4	4.00	2.67	2.33	2.67

**Table 5:** Reactome based statistical ranking for shared information methods organized by term similarity type and ontology. Methods showing a higher VI similarity to Reactome pathways have lower rank. Bold font represents the best average rank for each category.

	Biological process				Molecular function				Cellular component				Mean by term similarity		
Method	Resnik	Lin	JC	Mean	Resnik	Lin	JC	Mean	Resnik	Lin	JC	Mean	Resnik	Lin	JC
CASI	5	1	1	2.33	1	1	1	**1.00**	5	1	1	2.33	3.67	**1.00**	**1.00**
MICA	1	1	1	**1.00**	1	1	1	**1.00**	4	5	5	4.67	2.00	2.33	2.33
GraSM	1	1	1	**1.00**	1	1	1	**1.00**	1	1	1	**1.00**	**1.00**	**1.00**	**1.00**
A-GraSM	1	1	1	**1.00**	1	1	1	**1.00**	1	1	1	**1.00**	**1.00**	**1.00**	**1.00**
SF	1	1	1	**1.00**	1	1	1	**1.00**	1	1	1	**1.00**	**1.00**	**1.00**	**1.00**

**Table 6:** Protein interaction prediction ranking for shared information methods organized by term similarity type and ontology. Rankings were established by comparisons based on the area under the ROC curve calculated from bootstrap samples. Methods with greater AUC scores have lower rank. Bold font represents the best average rank for each category.

	Biological process				Molecular function				Cellular component				Mean by term similarity		
Method	Resnik	Lin	JC	Mean	Resnik	Lin	JC	Mean	Resnik	Lin	JC	Mean	Resnik	Lin	JC
CASI	3	1	4	2.67	5	1	4	3.33	5	2	4	3.67	4.33	**1.33**	4.00
MICA	1	1	1	**1.00**	4	4	1	3.00	4	5	1	3.33	3.00	3.33	**1.00**
GraSM	2	3	4	3.00	1	4	4	3.00	3	2	4	3.00	**2.00**	3.00	4.00
A-GraSM	3	3	3	3.00	1	1	1	**1.00**	2	2	3	2.33	**2.00**	2.00	2.33
SF	3	3	2	2.67	3	1	1	1.67	1	1	2	**1.33**	2.33	1.67	1.67

## Appendix A Detailed description of shared information algorithms

### A.1 Common ancestor shared information

Common ancestor shared information (CASI) is a naive baseline algorithms that calculated the simple mean IC of all common ancestors. Eq. (A.2) defines CASI where |*CA*(*t*_1_,*t*_2_)| is the number of shared ancestors.

(A.1) $$SI_{CASI}(t_{1},t_{2})=\frac{\sum_{t\in CA(t_{1},t_{2})}IC(t)}{\left|CA(t_{1},t_{2})\right|}$$

### A.2 Most informative common ancestor shared information

Most informative common ancestor (MICA) shared information is equivalent to Resnik term similarity and is defined by the IC of minimum subsumer. MICA shared information is completely described in the main text above through Eqs. (3), (4), and (5). The MICA represents the most specific concept that contains both *t*_1_ and *t*_2_ as descendants.

### A.3 Couto et al. 2007, GraSM

The graph-based semantic measure or GraSM was developed by Couto et al. [20]. GraSM calculates the set of disjunctive common ancestors (DCA) and computes their average as the shared information. Eq. (A.2) defines the disjunctive ancestor pairs for a term *t* where *a*_1_ and *a*_2_ are ancestor terms and *Paths* represents the number of unique paths from the ancestor to the descendant [49]. Couto et al. [20] give an algorithm for calculating if a pair of ancestor terms (*a*_1_,*a*_2_) are disjunctive in a descendant term *t* reproduced here in Algorithm 1. From this definition, the DCA set is defined in Eq. (A.3). Finally, the shared information is calculated as the mean IC of the set of *DCA*(*t*_1_,*t*_2_) in Eq. (A.4).

(A.2) $$\begin{array}{ll} DA(t)=\{(a_{1},a_{2})| & (\exists p:p\in \text{Paths}(a_{1},t)\wedge a_{2}\notin p)\wedge \\ & \left(\exists p:p\in \text{Paths}(a_{2},t)\wedge a_{1}\notin p)\right\} \end{array}$$

Algorithm 1 *DisjAnc*(*t*,(*a*_1_,*a*_2_))

(A.3) $$\begin{array}{ll} DCA(t_{1},t_{2})= & \left\{a_{1}|a_{1}\in CA(t_{1},t_{2})\wedge \right)\\ & \forall a_{2}:(a_{2}\in CA(t_{1},t_{2})\wedge IC(a_{1})\leq IC(a_{2})\wedge a_{1}\neq a_{2})\\ & \left(\;⟹\;(a_{1},a_{2})\in DA(t_{1})\cup DA(t_{2})\right\} \end{array}$$

(A.4) $$SI_{GraSM}(t_{1},t_{2})=\frac{\sum_{t\in DCA(t_{1},t_{2})}IC(t)}{\left|DCA(t_{1},t_{2})\right|}$$

### A.4 Adjusted GraSM

Adjusted GraSM makes only a slight change to the original algorithm. Changing the *greater than or equal to* (≥) in Algorithm 1 to a *strictly greater than* ( >) in the path number comparison causes the algorithm's behavior to change, and the size of the DCA set is reduced. Supplemental Information Trace 1 and 2 provided a detailed example of the differences between these two algorithms on a small calculation.

### A.5 Semantic frontier

The semantic frontier (SF) algorithm was developed to address the potential under estimation of shared information and costly runtime of GraSM. The SF algorithm calculates shared information by averaging the IC of concepts that form the border between the common ancestor set and ancestors unique to each term. We term these concepts the *semantic frontier* because they represent different borders to a shared territory. The SF calculation is outlined in Algorithms 2 and 3. The *target* function in Algorithm 3 returns the destination node of a directed edge. The SF algorithm is based on the concept of a breadth first search (BFS) visitor. A BFS visitor performs actions when certain events in a BFS occur. The SF algorithm constructs a BFS visitor that starts at a specific term in the GO graph and visits all ancestor terms on its way to the ontology root. Anytime a new edge is visited, it is added to the *visitedEdges* set for the starting term. Two sets are constructed in this way (*visitedEdgesT*1 and *visitedEdgesT*2) for the terms of interest *t*_1_ and *t*_2_. Any term *t* that satisfies Eq. (A.5) is a member of the semantic frontier of *t*_1_ and *t*_2_.

Algorithm 2 *SemanticFrontier*(*t*_1_,*t*_2_)

Algorithm 3 *V isitedEdgesBFS*(*t*,*visitedEdges*,*IncidentEdges*)

(A.5) $$\begin{array}{ll} t\in CA(t_{1},t_{2})\wedge \exists e| & target(e)=t\wedge \\ & ((e\in visitedEdgesT1\wedge e\notin visitedEdgesT2)\vee \\ & \left(e\in visitedEdgesT2\wedge e\notin visitedEdgesT1)\right) \end{array}$$

The SF algorithm is an efficient implementation of an algorithm developed by Zhang and Lai [21] that they call exclusively inherited shared information (EISI). The shared information of these two equivalent methods is defined in Eq. (A.6) where *SF*(*t*_1_,*t*_2_) represents the set of terms in the semantic frontier (or exclusively inherited common ancestors). Although equivalent in runtime complexity to EISI, the SF algorithm uses an edge visitor to construct only the necessary set of terms and descendants. This leads to a reduction in the search space as well as more opportunities for early exit. A side-by-side runtime comparison of the two algorithms was conducted, but neither algorithm showed a consistent and repeatable speed advantage (data not shown). As the size of the GO graph continues to grow, the SF algorithm may offer more of an advantage through reduction of the search space.

(A.6) $$SI_{SF}(t_{1},t_{2})=SI_{EISI}(t_{1},t_{2})=\frac{\sum_{t\in SF(t_{1},t_{2})}IC(t)}{\left|SF(t_{1},t_{2})\right|}$$

## Appendix B Supplementary data

Supplementary material 1: Supplemental figures and methods.

Supplementary material 2: Short execution traces of GraSM and A-GraSM that illustrate behavior described in the text.

Table S1 Build times for the calculation of term-to-term similarity matrices. Table S1

Table S2 The complete results for the RRBS dataset showing mean Spearman correlation and standard deviations. Table S2

Table S3 The complete results for the Jaccard index showing mean Spearman correlation and standard deviations. Table S3

Table S4 The complete results for the TF * IDF Pfam similarity showing mean Spearman correlation and standard deviations. Table S4

Table S5 The complete results for the gene expression correlation (gene-to-gene Pearson exp. corr.) showing mean Spearman correlation and standard deviations. Table S5

Table S6 The complete results for the gene expression correlation (gene-to-gene Spearman exp. corr.) showing mean Spearman correlation and standard deviations. Table S6

Table S7 The complete results for Reactome clustering. This table provides all mean VI values and standard deviations for all algorithms. Table S7

Table S8 The complete results for PPI prediction data. This table provides all mean ROC AUC values and standard deviations for all algorithms. Table S8
