# Supplementary material for: The effects of shared information on semantic calculations in the gene ontology
Source: Comput Struct Biotechnol J. 2017 Jan 30;15:195–211. doi: 10.1016/j.csbj.2017.01.009 (PMC5299144; doi:10.1016/j.csbj.2017.01.009)
Supplement: Supplementary file 1 — Supplementary material 1: Supplemental figures and methods. [file mmc1.doc]

**Supplementary Information**

The effect of shared information on semantic calculations in the Gene Ontology

Paul W. Bible, Hong-Wei Sun, Maria I. Morasso, Rasiah Loganantharaj, and Lai Wei

Extended Results Section

**CESSM**

The CESSM dataset uses 1039 heavily researched proteins from 63 species and is comprised 13430 protein pairs . In **Supplemental Figure S1-S3**, the performance for three aggregation methods and four SI methods are presented for each Ontology type. The MICA shared information is omitted as the data for that measure is readily available at the CESSM site.

**Supplemental Figure S1.** The CESSM benchmark comparing shared information (SI) algorithm shows that BMA appears to outperform other measures using biological process (BP) annotations. When using Resnik, BMA and max appear to offer similar performance for CESSM.


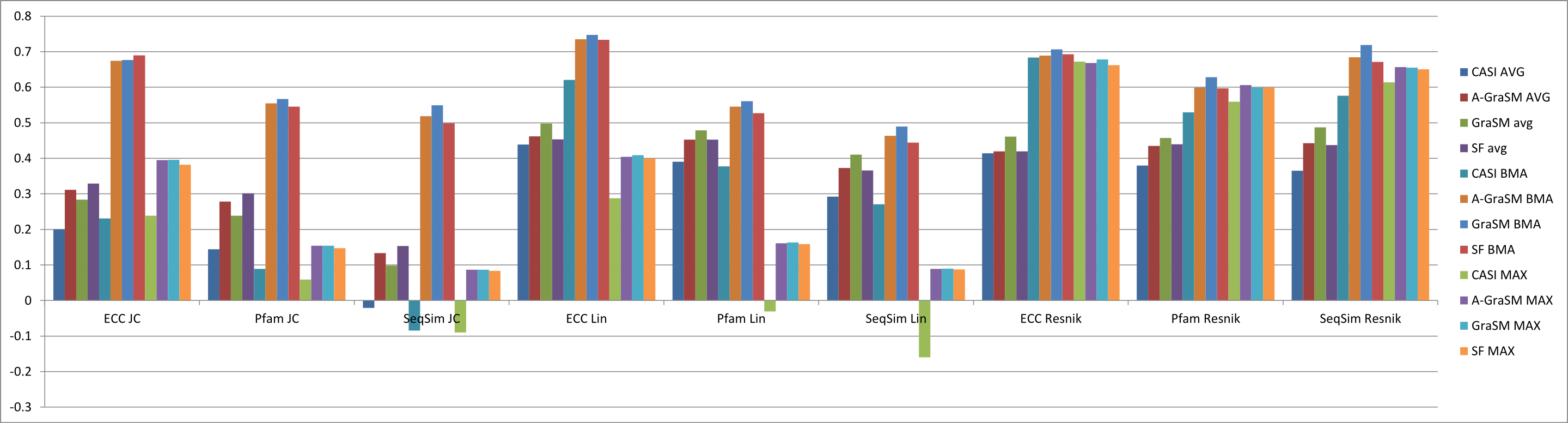


**Supplemental Figure S2.** The CESSM benchmark comparing shared information (SI) algorithm shows that BMA appears to outperform other measures using molecular function (MF) annotations. When using Resnik, BMA and max appear to offer similar performance for CESSM.


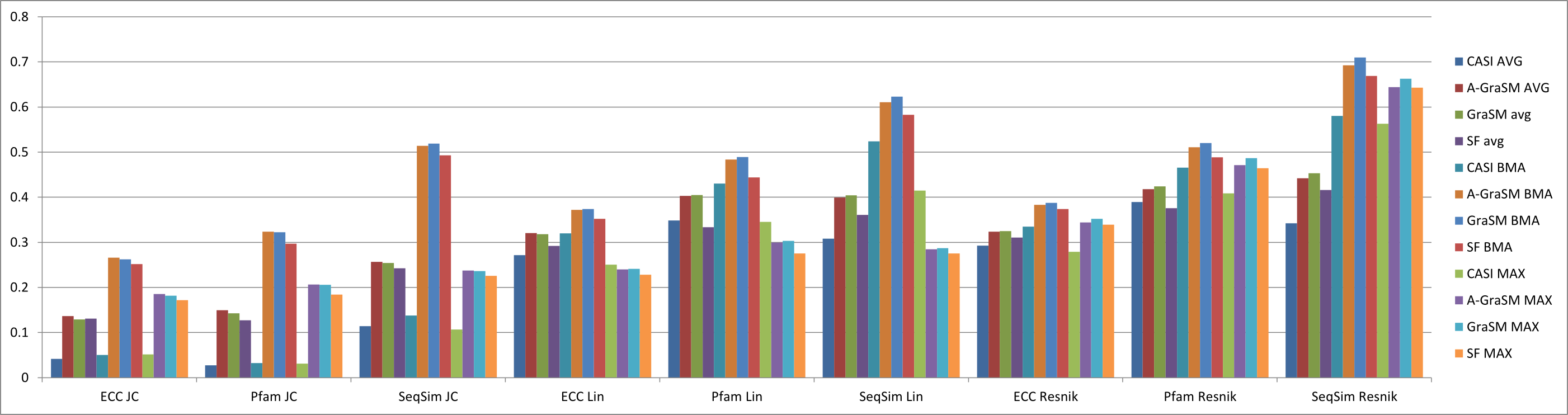


**Supplemental Figure S3.** The CESSM benchmark comparing shared information (SI) algorithm shows that BMA appears to outperform other measures using cellular component (CC) annotations. When using Resnik, BMA and max appear to offer similar performance for CESSM.

**Expression Correlation with Semantic Similarity**

As reported by Xu et al. (2009), when genes are binned by expression correlation the correlation with semantic measures increases. Figure S4 shows this same pattern using the BP Jiang-Conrath A-GraSM measure.

**Supplemental Figure S4**. Correlation between semantic similarity and expression correlation. As genes are binned by their expression correlation, their expression and semantic similarity show greater correlation.

**Table S1.** Build times for the calculation of term-to-term similarity matrices.

**Table S2.** The complete results for the RRBS dataset showing mean Spearman correlation and Standard deviations.

**Table S3** The complete results for the Jaccard index showing mean Spearman correlation and Standard deviations.

**Table S4** The complete results for the TF*IDF Pfam similarity showing mean Spearman correlation and Standard deviations.

**Table S5** The complete results for the gene expression correlation (gene-to-gene Pearson exp. corr.) showing mean Spearman correlation and Standard deviations.

**Table S6** The complete results for the gene expression correlation (gene-to-gene Spearman exp. corr.) showing mean Spearman correlation and Standard deviations.

**Table S7** The complete results for Reactome clustering. This table provides all mean VI values and standard deviations for all algorithms.

**Table S8** The complete results for PPI prediction data. This table provides all mean ROC AUC values and standard deviations for all algorithms.

References

Pesquita, C.*, et al.* Metrics for GO based protein semantic similarity: a systematic evaluation. *BMC bioinformatics* 2008;9 Suppl 5:S4.

Pesquita, C.*, et al.* CESSM: Collaborative evaluation of semantic similarity measures. *JB2009: Challenges in Bioinformatics* 2009;157.

Xu, Y.*, et al.* A novel insight into Gene Ontology semantic similarity. *Genomics* 2013;101(6):368-375
