# Supplementary material for: The effects of shared information on semantic calculations in the gene ontology
Source: Comput Struct Biotechnol J. 2017 Jan 30;15:195–211. doi: 10.1016/j.csbj.2017.01.009 (PMC5299144; doi:10.1016/j.csbj.2017.01.009)
Supplement: Supplementary file 2 — Supplementary material 2: Short execution traces of GraSM and A-GraSM that illustrate behavior described in the text. [file mmc2.docx]

**Trace 1**. A trace illustrating how the GraSM algorithm includes the ontology root due to the use of a *greater than or equal to* (≥) comparison.

The GraSM algorithm described by Couto et al. 2007.

The following trace illustrates an issue with using greater than or equal to (≥) in the path number comparison.

Consider the two concepts, polysaccharide binding (GO:0030247) and oligosaccharide binding (GO:0070492), that have a graph structure equivalent to the example in the Couto et al. 2007 paper. Let the nodes of the subgraph induced by these two terms be relabeled (A, B, C, D, E, F) as below. For this trace we are simply concerned with the calculation of the set of common disjunctive ancestors (CDA) of Share_GraSM_(c1,c2), where the concepts are nodes E (GO:0070492) and F (GO:0030247). Calculation of the average information content from this set is trivial.


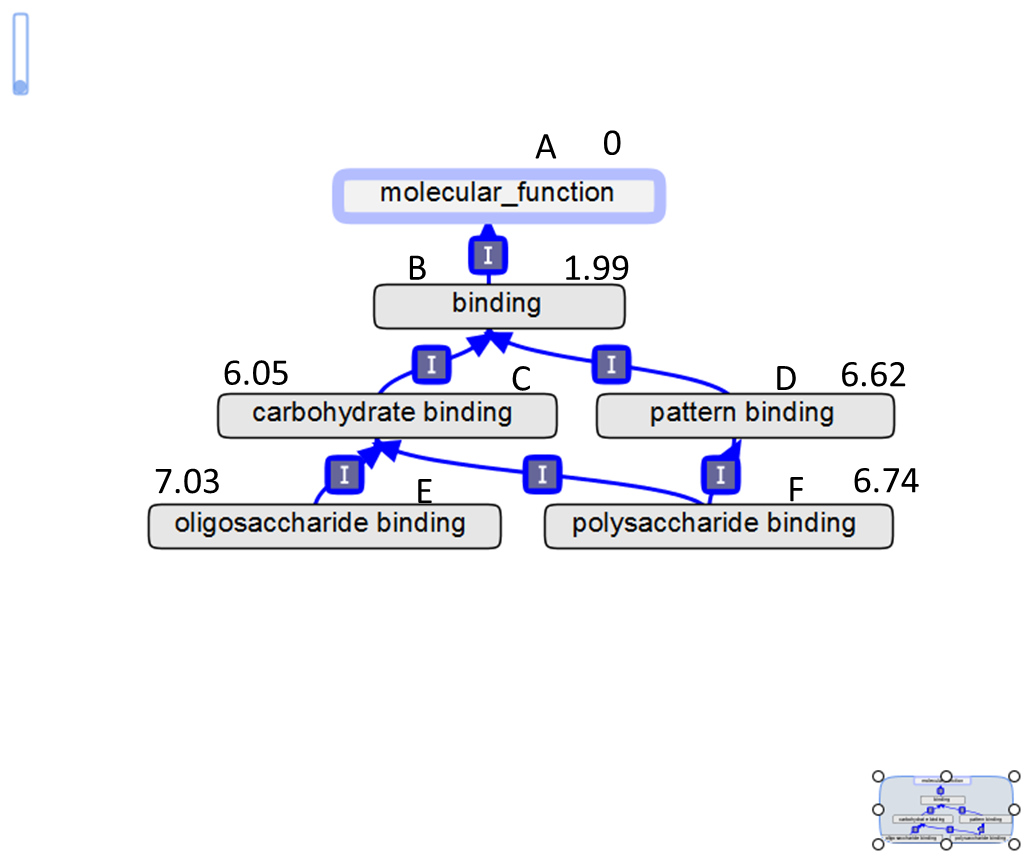


GO graph version available at <ftp://ftp.geneontology.org/go/ontology-archive/gene_ontology_edit.obo.2013-04-01.gz>. April 2013 version. Information content calculated with GGTK using mouse annotations from GOA at <ftp://ftp.ebi.ac.uk/pub/databases/GO/goa/old/MOUSE/gene_association.goa_mouse.108.gz>.

In the following trace, line numbers of the form ‘#)’ indicate the line of execution in the above algorithm. Entry into a subroutine and other comments are marked with *’s. The contents of collections are show to the right of the executed line. Implied variable assignments are show on a new line.

**Trace of ShareGraSM( E, F)**

1) Anc = CommonAncestors( E, F), Anc = {A, B, C}

2) CommonDisjAnc = {}

3) for all a in sortDescByIC(Anc), sortDescByIC(Anc) = {C, B, A}

a = C

4) isDisj = true

5) For all cda in CommonDisjAnc, CommonDisjAnc = {}

* Does not loop because CommonDisjAnc is empty *

8) if isDisj then

9) addTo(CommonDisjAnc, C), CommonDisjAnc = {C}

* for all a in sortDescByIC(Anc) *

a = B

4) isDisj = true

5) For all cda in CommonDisjAnc, CommonDisjAnc = {C}

6) isDisj = isDisj & ( DisjAnc(E (B, C)) or DisjAnc(F , (B, C)) )

* Entering Subroutine: DisjAnc(E , (B, C)) *

1) Require IC(B) ≤ IC(C), IC(B) = 1.99, IC(C) = 6.05, (IC(B) ≤ IC(C)) = True

2) nPaths = |Paths(B, C)| = 1

3) nPaths1 = |Paths(B, E)| = 1

4) nPaths2 = |Paths(C, E)| = 1

5) return nPaths1 ≥ nPaths * nPaths2, 1 ≥ 1*1 = True

*Because of ‘or’, no need to evaluate DisjAnc(F, (B, C)) *

*Return to line 6*

6) isDisj = isDisj & ( DisjAnc(E (B, C)) or DisjAnc(F , (B, C)) ), isDisj = True & (True or [not used]) = True

8) if isDisj then, isDisj = True, B is added

9) addTo(CommonDisjAnc, B), CommonDisjAnc = {C, B}

* for all a in sortDescByIC(Anc) *

a = A

4) isDisj = true

5) For all cda in CommonDisjAnc, CommonDisjAnc = {C, B}

cda = C

6) isDisj = isDisj & ( DisjAnc(E, (A, C)) or DisjAnc(F , (A, C)) )

* Entering Subroutine: DisjAnc(E, (A, C)) *

1) Require IC(A) ≤ IC(C), IC(A) = 0, IC(C) = 6.05, (IC(A) ≤ IC(C)) = True

2) nPaths = |Paths(A, C)| = 1

3) nPaths1 = |Paths(A, E)| = 1

4) nPaths2 = |Paths(C, E)| = 1

5) return nPaths1 ≥ nPaths * nPaths2, 1 ≥ 1*1 = True

*Because of ‘or’, no need to evaluate DisjAnc(F , (A, C))*

6) isDisj = isDisj & ( DisjAnc(E, (A, C)) or DisjAnc(F , (A, C)) ), isDisj = True & (True or [ not used]) = True

* loop returns to line (5)*

5) For all cda in CommonDisjAnc, CommonDisjAnc = {C, B}

cda = B

6) isDisj = isDisj & ( DisjAnc(E, (A, B)) or DisjAnc(F , (A, B)) )

* Entering Subroutine: DisjAnc(E, (A, B)) *

1) Require IC(A) ≤ IC(B), IC(A) = 0, IC(B) = 1.99, (IC(A) ≤ IC(B)) = True

2) nPaths = |Paths(A, B)| = 1

3) nPaths1 = |Paths(A, E)| = 1

4) nPaths2 = |Paths(B, E)| = 1

5) return nPaths1 ≥ nPaths * nPaths2, 1 ≥ 1*1 = True

*Return to line 6, Because of ‘or’, no need to evaluate DisjAnc(F , (A, C))*

6) isDisj = isDisj & ( DisjAnc(E, (A, B)) or DisjAnc(F , (A, B)) ), isDisj = True & (True or [ not used]) = True

8) if isDisj then,

9) addTo(CommonDisjAnc, A), CommonDisjAnc = {C, B, A}

*The root node (A) is added to the common disjoint ancestors*

**Trace of ShareGraSM( E, F) with strictly greater than (>) used in the DisjAnc subroutine. (Adjusted GraSM)**

1) Anc = CommonAncestors( E, F), Anc = {A, B, C}

2) CommonDisjAnc = {}

3) for all a in sortDescByIC(Anc), sortDescByIC(Anc) = { C, B, A }

a = C

4) isDisj = true

5) For all cda in CommonDisjAnc, CommonDisjAnc = {}

* Does not loop because CommonDisjAnc is empty *

8) if isDisj then

9) addTo(CommonDisjAnc, C), CommonDisjAnc = {C}

* for all a in sortDescByIC(Anc) *

a = B

4) isDisj = true

5) For all cda in CommonDisjAnc, CommonDisjAnc = {C}

6) isDisj = isDisj & ( DisjAnc(E (B, C)) or DisjAnc(F , (B, C)) )

* Entering Subroutine: DisjAnc(E , (B, C)) *

1) Require IC(B) ≤ IC(C), IC(B) = 1.99, IC(C) = 6.05, (IC(B) ≤ IC(C)) = True

2) nPaths = |Paths(B, C)| = 1

3) nPaths1 = |Paths(B, E)| = 1

4) nPaths2 = |Paths(C, E)| = 1

5) return nPaths1 > nPaths * nPaths2, 1 **>** 1*1 = False

* Entering Subroutine: DisjAnc(F , (B, C)) *

1) Require IC(B) ≤ IC(C), IC(B) = 1.99, IC(C) = 6.05, (IC(B) ≤ IC(C)) = True

2) nPaths = |Paths(B, C)| = 1

3) nPaths1 = |Paths(B, F)| = 2

4) nPaths2 = |Paths(C, F)| = 1

5) return nPaths1 > nPaths * nPaths2, 2 **>** 1*1 = True

*Return to line 6*

6) isDisj = isDisj & ( DisjAnc(E (B, C)) or DisjAnc(F , (B, C)) ), isDisj = True & (False or True) = True

8) if isDisj then, isDisj = True, B is added

9) addTo(CommonDisjAnc, B), CommonDisjAnc = {C, B}

* for all a in sortDescByIC(Anc) *

a = A

4) isDisj = true

5) For all cda in CommonDisjAnc, CommonDisjAnc = {C, B}

cda = C

6) isDisj = isDisj & ( DisjAnc(E, (A, C)) or DisjAnc(F , (A, C)) )

* Entering Subroutine: DisjAnc(E, (A, C)) *

1) Require IC(A) ≤ IC(C), IC(A) = 0, IC(C) = 6.05, (IC(A) ≤ IC(C)) = True

2) nPaths = |Paths(A, C)| = 1

3) nPaths1 = |Paths(A, E)| = 1

4) nPaths2 = |Paths(C, E)| = 1

5) return nPaths1 > nPaths * nPaths2, 1 > 1*1 = False

* Entering Subroutine: DisjAnc(F, (A, C)) *

1) Require IC(A) ≤ IC(C), IC(A) = 0, IC(C) = 6.05, (IC(A) ≤ IC(C)) = True

2) nPaths = |Paths(A, C)| = 1

3) nPaths1 = |Paths(A, F)| = 2

4) nPaths2 = |Paths(C, F)| = 1

5) return nPaths1 > nPaths * nPaths2, 2 > 1*1 = True

*Return to line 6*

6) isDisj = isDisj & ( DisjAnc(E, (A, B)) or DisjAnc(F , (A, B)) ), isDisj = True & (False or True) = True

*Return to Line 5*

5) For all cda in CommonDisjAnc, CommonDisjAnc = {C, B}

cda = B

6) isDisj = isDisj & ( DisjAnc(E, (A, B)) or DisjAnc(F , (A, B)) )

* Entering Subroutine: DisjAnc(E, (A, B)) *

1) Require IC(A) ≤ IC(B), IC(A) = 0, IC(B) = 1.99, (IC(A) ≤ IC(B)) = True

2) nPaths = |Paths(A, B)| = 1

3) nPaths1 = |Paths(A, E)| = 1

4) nPaths2 = |Paths(B, E)| = 1

5) return nPaths1 > nPaths * nPaths2, 1 > 1*1 = False

* Entering Subroutine: DisjAnc(F, (A, B)) *

1) Require IC(A) ≤ IC(B), IC(A) = 0, IC(B) = 1.99, (IC(A) ≤ IC(B)) = True

2) nPaths = |Paths(A, B)| = 1

3) nPaths1 = |Paths(A, F)| = 2

4) nPaths2 = |Paths(B, F)| = 2

5) return nPaths1 > nPaths * nPaths2, 2 > 1*2 = False

*Return to line 6*

6) isDisj = isDisj & ( DisjAnc(E, (A, B)) or DisjAnc(F , (A, B)) ), isDisj = True & (False or False) = False

8) if isDisj then, isDisj = False, A is not added.

*The root node is not added to the CDA set when *strictly greater than* (>) is used*

Trivially calculate the mean information content of the CDA set.

These two traces illustrate that modifying the comparison in the DisjAnc subroutine, results in different behavior that changes the shared information content calculation.

**Trace 2.** A trace illustrating a case where the Adjusted GraSM measure includes the root node.

Although Adjusted GraSM typically reduces the CDA set, there are cases where the root node is included in the CDA set. The following trace illustrates once such case.

Consider the two GO concepts, electron transport chain (GO:0022900) and cellular macromolecule metabolic process (GO:0044260), with the following subgraph:


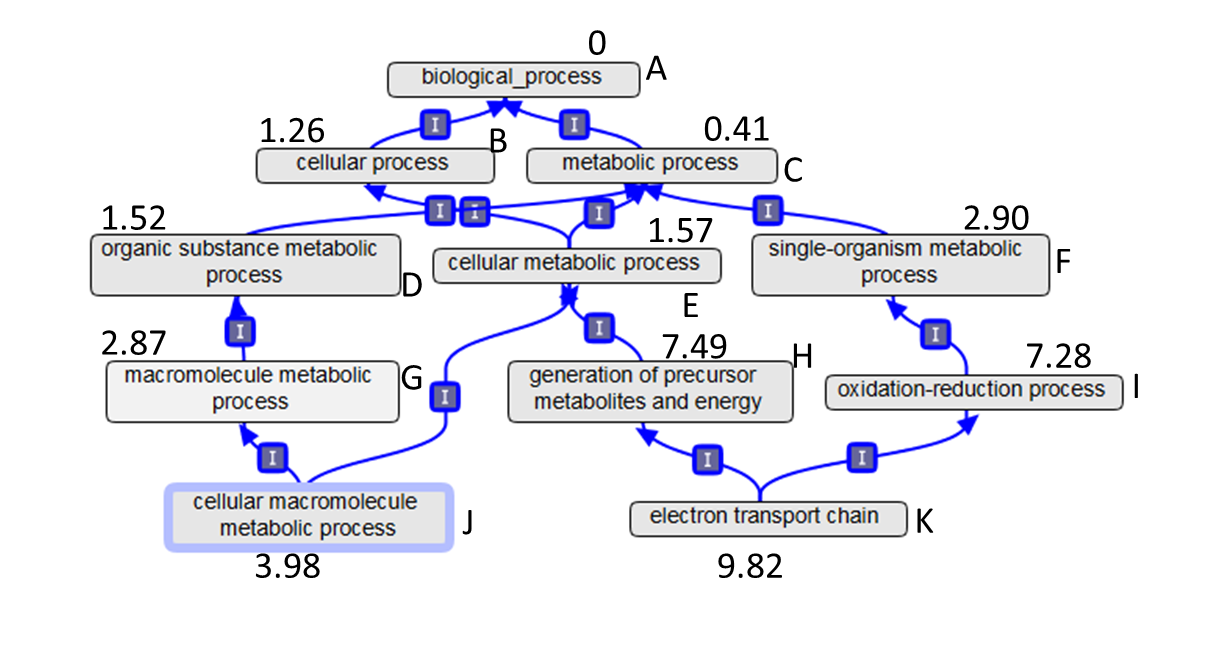


GO graph version available at <ftp://ftp.geneontology.org/go/ontology-archive/gene_ontology_edit.obo.2013-04-01.gz>. April 2013 version. Information content calculated with GGTK using mouse annotations from GOA at <ftp://ftp.ebi.ac.uk/pub/databases/GO/goa/old/MOUSE/gene_association.goa_mouse.108.gz>.

For this trace we are simply concerned with the calculation of the set of common disjoint ancestors of ShareGraSM(c1,c2). Calculation of the average information content from this set is trivial.

We are concerned with ShareGraSM(GO:0022900, GO:0044260).

Let the nodes of the subgraph be re-labelled (A, B, C, D, E, F, G, H, I, J, K) as above.

Trace of ShareGraSM( J, K)

1) Anc = CommonAncestors( J, K), Anc = {A, B, C, E}

2) CommonDisjAnc = {}

3) for all a in sortDescByIC(Anc), sortDescByIC(Anc) = {E, B, C, A}

a = E

4) isDisj = true

5) For all cda in CommonDisjAnc, CommonDisjAnc = {}

* Does not loop because CommonDisjAnc is empty *

8) if isDisj then

9) addTo(CommonDisjAnc, E), CommonDisjAnc = {E}

* for all a in sortDescByIC(Anc) *

a = B

4) isDisj = true

5) For all cda in CommonDisjAnc, CommonDisjAnc = {E}

6) isDisj = isDisj & ( DisjAnc(J , (B, E)) or DisjAnc(K , (B, E)) )

* Entering Subroutine: DisjAnc(J , (B, E)) *

1) Require IC(B) ≤ IC(E), IC(B) = 1.26, IC(E) = 1.57, (IC(B) ≤ IC(E)) = True

2) nPaths = |Paths(B, E)| = 1

3) nPaths1 = |Paths(B, J)| = 1

4) nPaths2 = |Paths(E, J)| = 1

5) return nPaths1 > nPaths * nPaths2, 1 > 1*1 = False

* Entering Subroutine: DisjAnc(K , (B, E)) *

1) Require IC(B) ≤ IC(E), IC(B) = 1.26, IC(E) = 1.57, (IC(B) ≤ IC(E)) = True

2) nPaths = |Paths(B, E)| = 1

3) nPaths1 = |Paths(B, K)| = 1

4) nPaths2 = |Paths(E, K)| = 1

5) return nPaths1 > nPaths * nPaths2, 1 > 1*1 = False

*Return to line 6*

6) isDisj = isDisj & ( DisjAnc(J , (B, E)) or DisjAnc(K , (B, E)) ), isDisj = True & (False or False) = False

8) if isDisj then, isDisj = False, B is not added

* for all a in sortDescByIC(Anc) *

a = C

4) isDisj = true

5) For all cda in CommonDisjAnc, CommonDisjAnc = {E}

6) isDisj = isDisj & ( DisjAnc(J , (C, E)) or DisjAnc(K , (C, E)) )

* Entering Subroutine: DisjAnc(J , (C, E)) *

1) Require IC(C) ≤ IC(E), IC(C) = 0.41, IC(E) = 1.57, (IC(C) ≤ IC(E)) = True

2) nPaths = |Paths(C, E)| = 1

3) nPaths1 = |Paths(C, J)| = 1

4) nPaths2 = |Paths(E, J)| = 1

5) return nPaths1 > nPaths * nPaths2, 1 > 1*1 = False

* Entering Subroutine: DisjAnc(K , (C, E)) *

1) Require IC(C) ≤ IC(E), IC(C) = 0.41, IC(E) = 1.57, (IC(C) ≤ IC(E)) = True

2) nPaths = |Paths(C, E)| = 1

3) nPaths1 = |Paths(C, K)| = 2

4) nPaths2 = |Paths(E, K)| = 1

5) return nPaths1 > nPaths * nPaths2, 2 > 1*1 = True

*Return to line 6*

6) isDisj = isDisj & ( DisjAnc(J , (C, E)) or DisjAnc(K , (C, E)) ) isDisj = True & (False or True) = True

8) if isDisj then,

9) addTo(CommonDisjAnc, C), CommonDisjAnc = {E, C}

* for all a in sortDescByIC(Anc) *

a = A

4) isDisj = true

5) For all cda in CommonDisjAnc, CommonDisjAnc = {E, C}

cda = E

6) isDisj = isDisj & ( DisjAnc(J , (A, E)) or DisjAnc(K , (A, E)) )

* Entering Subroutine: DisjAnc(J , (A, E)) *

1) Require IC(A) ≤ IC(E), IC(A) = 0, IC(E) = 1.57, (IC(A) ≤ IC(E)) = True

2) nPaths = |Paths(A, E)| = 2

3) nPaths1 = |Paths(A, J)| = 3

4) nPaths2 = |Paths(E, J)| = 1

5) return nPaths1 > nPaths * nPaths2, 3 > 2*1 = True

*Because of ‘or’, no need to evaluate DisjAnc(K , (A, E)) *

*Return to line 6*

6) isDisj = isDisj & ( DisjAnc(J , (A, E)) or DisjAnc(K , (A, E)) ) isDisj = True & (True or [ not used]) = True

* For all cda in CommonDisjAnc *

cda = C

6) isDisj = isDisj & ( DisjAnc(J , (A, C)) or DisjAnc(K , (A, C)) )

* Entering Subroutine: DisjAnc(J , (A, C)) *

1) Require IC(A) ≤ IC(C), IC(A) = 0, IC(C) = 0.41, (IC(A) ≤ IC(C)) = True

2) nPaths = |Paths(A, C)| = 1

3) nPaths1 = |Paths(A, J)| = 3

4) nPaths2 = |Paths(C, J)| = 2

5) return nPaths1 > nPaths * nPaths2, 3 > 1*2 = True

*Because of ‘or’, no need to evaluate DisjAnc(K , (A, C)) *

*Return to line 6*

6) isDisj = isDisj & ( DisjAnc(J , (A, C)) or DisjAnc(K , (A, C)) ), isDisj = True & (True or [ not used]) = True

8) if isDisj then,

9) addTo(CommonDisjAnc, A), CommonDisjAnc = {E, C, A}

*The root node is added to the common disjoint ancestors*

…

Trivially calculate average IC of CommonDisjAnc and return.
